# An integration of genome-wide survey, homologous comparison and gene expression analysis provides a basic framework for the ZRT, IRT-like protein (ZIP) in foxtail millet

**DOI:** 10.3389/fpls.2024.1467015

**Published:** 2024-09-05

**Authors:** Jie Zheng, Yunxiao Ma, Yu Liang, Tianhan Zhang, Chang Chen, Aduragbemi Amo, Wenyu Wang, Fangfang Ma, Yuanhuai Han, Hongying Li, Siyu Hou, Yang Yang

**Affiliations:** ^1^ College of Agriculture, Houji Laboratory of Shanxi Province, Shanxi Agricultural University, Taiyuan, Shanxi, China; ^2^ Department of Horticultural Sciences, Texas A&M University, College Station, TX, United States; ^3^ Texas A&M AgriLife Research and Extension Center, Weslaco, TX, United States; ^4^ Xinjiang Research Institute, Join Hope Seed Co., Ltd, Changji, Xinjiang, China

**Keywords:** foxtail millet, ZIP transporter, homologous comparison, expression characteristics, functional prediction

## Abstract

Essential mineral elements such as zinc and iron play a crucial role in maintaining crop growth and development, as well as ensuring human health. Foxtail millet is an ancient food crop rich in mineral elements and constitutes an important dietary supplement for nutrient-deficient populations. The ZIP (ZRT, IRT-like protein) transporters are primarily responsible for the absorption, transportation and accumulation of Zn, Fe and other metal ions in plants. Here, we identified 14 ZIP transporters in foxtail millet (SiZIP) and systematically characterized their phylogenetic relationships, expression characteristics, sequence variations, and responses to various abiotic stresses. As a result, SiZIPs display rich spatiotemporal expression characteristics in foxtail millet. Multiple SiZIPs demonstrated significant responses to Fe, Cd, Na, and K metal ions, as well as drought and cold stresses. Based on homologous comparisons, expression characteristics and previous studies, the functions of SiZIPs were predicted as being classified into several categories: absorption/efflux, transport/distribution and accumulation of metal ions. Simultaneously, a schematic diagram of SiZIP was drawn. In general, SiZIPs have diverse functions and extensively involve in the transport of metal ions and osmotic regulation under abiotic stresses. This work provides a fundamental framework for the transport and accumulation of mineral elements and will facilitate the quality improvement of foxtail millet.

## 1 Introduction

As essential mineral elements, iron (Fe) and zinc (Zn) exert a significant role in sustaining the growth, development, and physiological metabolism of plants. Zn serves as a cofactor for over 300 enzymes, encompassing zinc finger binding protein, ring finger protein, single serine protein kinase, etc., and acts as a binding factor for the normal functionalities of more than 2000 transcription factors, influencing biological metabolism, biomembrane stability, and gene expression (Coleman, 1998). Zn deficiency can have a substantial impact on plant growth and development, such as inducing oxidative damage (Cakmak, 2000); inhibiting auxin synthesis (Broadley et al., 2012); reducing RNA polymerase activity, decreasing the number of ribosomes, and lowering the rate and content of protein synthesis (Ho, 2004). Fe, existing in two ionic forms of Fe^2+^ and Fe^3+^, participates in the synthesis of chlorophyll precursor ALA (δ-Aminolevulinic Acid) (Pushnik et al., 1984) and the synthesis of crucial plant proteins like Fe-S proteins (Rouault, 2015), which are indispensable for maintaining the normal operation of various physiological processes in plants, including photosynthesis, respiration, and nitrogen fixation (Broadley et al., 2012). A moderate increase of Zn and Fe in crops has a positive influence on yield and quality, while their deficiency can result in stunted plants and a considerable reduction in the Zn and Fe content of grains (Sadeghzadeh, 2013; Pinson et al., 2015).

In addition to being significant mineral elements that directly impact plant growth and development, Zn and Fe are also indispensable trace elements for humans, and their accumulation within the body is closely associated with human health. More than half of the global population has a seriously insufficient intake of Fe, Zn, and selenium (Se) (White and Broadley, 2009; Trijatmiko et al., 2016). The ‘Hidden Hunger’ caused by the deficiency of essential vitamins and micronutrients has affected two billion people worldwide and emerges as a global challenge (Khush et al., 2012). Insufficient intake of essential micronutrients, aside from causing a weakened immune system, also leads to poor physical and mental development in children and even fatal diseases (Bourke et al., 2016). Moreover, with the advancement of industry and agriculture, such as mining, metal smelting and excessive application of pesticides and fertilizers, there is severe heavy metal pollution such as cadmium (Cd) in the soil, which can readily enter the human body through the food chain and directly threaten human health (Pan et al., 2016; Yang et al., 2021c). Due to the similar geochemical behavior of metal elements and the poor specificity of plant metal transporters, there exists a certain synergism/antagonism between trace metal elements and harmful heavy metal elements in the uptake, transport, and accumulation of crops (Bolan et al., 2003; Baxter, 2015; Wang et al., 2022).

The zinc-regulated transporters, iron-regulated transporter-like protein (ZIP) family acts as the primary functional proteins in plants responsible for absorbing Zn and Fe ions from the soil. It comprises two major categories of protein members: zinc-regulated transporter (ZRT) and iron-regulated transporter (IRT)-like proteins. Members of the ZIP family are widely distributed in various tissues of plants and play crucial roles in the absorption, transport, distribution and utilization of Zn and Fe ions (Eide, 2006). AtIRT1 was first discovered in *Arabidopsis* to specifically transport Fe and Zn ions in roots and was induced by Fe deficiency (Eide et al., 1996); Compared to the wild type, the *irt1* mutant displayed a 70% reduction in Fe content in leaves and exhibited a chlorotic phenotype (Vert et al., 2002), and overexpression of *AtIRT1* in the *irt1* mutant could alleviate the chlorotic phenotype (Krämer et al., 2007); AtIRT2 also demonstrated a similar function to AtIRT1 in transporting Fe (Vert et al., 2001). AtZIP1 and AtZIP2 are crucial for plants to absorb manganese (Mn) and Zn through the root system and transport them from the roots to the leaves (Milner et al., 2013). Although both are reported as major Zn transporter genes, their subcellular localizations and tissue expression patterns are slightly different, indicating that these two genes have distinct functions. *AtZIP*1 is primarily expressed on the tonoplast of leaf veins and root cells, while *AtZIP*2 is highly expressed on the plasma membrane of root column cells. AtZIP1 and AtZIP2 are respectively involved in the reactivation of metal ions from vacuoles to the cytoplasm and the absorption of Mn and Zn by roots, respectively (Milner et al., 2013). Other studies have also confirmed that *AtZIP* genes are induced by Zn deficiency, and some *AtZIP* genes are directly involved in the accumulation of Zn in the edible parts of plants (Ramegowda et al., 2013; Gaitan-Solis et al., 2015). In rice (*Oryza sativa*), both *OsIRT1* and *OsIRT2* are highly expressed in roots for Fe transport and are significantly induced by Fe deficiency (Li et al., 2019). Overexpression of both *OsIRT1* and *OsIRT2* enhances the resistance of plants to Fe deficiency stress and concurrently increases the content of Fe and Zn in grains (Bughio et al., 2002; Ishimaru et al., 2007a). Overexpression of *OsZIP1* increases the accumulation of Zn and Fe in roots, buds and seeds (Ishimaru et al., 2006), while overexpression of *OsZIP4* and *OsZIP5* significantly elevates the Zn content in roots of transgenic plants, but not in grains (Ishimaru et al., 2007b; Lee et al., 2010a). It has been verified that ZIP transporters are also involved in the transportation of bivalent metal cations such as Mn^2+^ and Cd^2+^ (Chen et al., 2008; Fan et al., 2021). For instance, the expression levels of *ZIP2* and *ZIP3* in Chinese cabbage (*Brassica chinensis*), as well as the expression level of *SlZIP4* in leaves of tomato (*Solanum lycopersicum*), are closely related to Cd transport and concentration (Yu et al., 2017; Wu et al., 2019). Generally, different ZIP transporters influence plant growth and development as well as the accumulation of trace elements in grains by mediating the absorption, transportation and distribution of divalent metal ions such as Fe and Zn. Therefore, studying the transportation and accumulation of metal ions such as Zn, Fe and Cd mediated by different ZIP transporters is crucial for improving food quality and ensuring human health.

Food crops are the main dietary source of energy and essential trace elements for human intake. Due to the relatively low contents of Zn and Fe in maize and rice, the risk of deficiency in nutritional elements such as Fe and Zn among people who mainly consume corn and rice, especially those in poverty-stricken areas with simple dietary structures, has significantly increased (Wessells and Brown, 2012). Currently, it is widely believed that the solutions to address the ‘hidden hunger’ caused by the deficiency of trace elements such as Zn and Fe in humans include measures such as dietary diversification, functional nutritional supplements for food, or biofortification of staple foods (Yan et al., 2023). Foxtail millet, as one of the earliest domesticated cereal crops by humans, is widely cultivated in arid and semi-arid regions of Asia and Africa, including China, India, and Nigeria. The hulled product of foxtail millet, known as millet, is rich in various nutrients such as amino acids, vitamins, and minerals that are essential for the human body. The contents of Fe and Zn are much higher than those of staple crops such as rice and maize (*Zea mays*), and it can serve as a good dietary supplement for people with nutrient deficiencies (He et al., 2022). However, the mechanism of nutrient enrichment is still unclear. Although ZIP transporters have been investigated in *Arabidopsis*, rice, maize and other staple crops, scarce knowledge is available regarding the ZIP transporters in foxtail millet (Eide et al., 1996; Eide, 2006; Ishimaru et al., 2006, 2007; Li et al., 2013). Here, we systematically identified and characterized ZIP transporters in foxtail millet using the latest genomic data, and predicted their functions using a strategy integrating homology comparison, expression characteristic analysis and previous studies. The goals of this study are to: (1) accurately identify and characterize the *ZIP* gene family in foxtail millet; (2) elucidate the characteristics of family expansion and functional divergence; (3) explore *SiZIP* expression patterns in different tissues and under metal ion and abiotic stresses; (4) clarify the variation characteristics of *SiZIPs* at the sequence and expression levels, and explore their values in breeding and improvement of foxtail millet; (5) predict the potential functions of *SiZIPs*. This study will lay the foundation for further functional research on foxtail millet *ZIP* genes, and will also contribute to the elucidation of the molecular mechanisms underlying metal ion transport and accumulation in foxtail millet.

## 2 Materials and methods

### 2.1 Identification and basic physicochemical characteristics of *SiZIP*s

The genomic sequence, protein sequence and genomic annotation file of the foxtail millet material ‘*xiaomi*’ were obtained from the Multi-omics Database for *Setaria italica* (MDSi, http://foxtail-millet.biocloud.net/) (Yang et al., 2020). The genomic information of barley (*Hordeum vulgare*), sorghum (*Sorghum bicolor*), maize, rice, *Arabidopsis*, and potato (*Solanum tuberosum*) was downloaded from the Ensemble Plants database (http://plants.ensembl.org/index.html). The ZIP proteins in barley were obtained from published papers and were used together with ZIP proteins from rice and *Arabidopsis* as seed sequences for BLASTP alignment (BLAST+, version 2.12.0) of the protein databases of foxtail millet, sorghum, maize, and potato (Deng et al., 2022). Protein sequences with a similarity greater than 50% and an e-value less than 10^-5^ were considered as candidates for ZIP. The conserved domain (PF02535) of all candidate genes was screened using the HMMsearch tool (version 3.3.2) with a threshold of an e-value less than 10^-10^, and only those candidate genes containing the complete domain were retained. The putative candidate *ZIPs* were obtained by manually removing different transcripts of the same gene. Subsequently, all *ZIPs* were renamed according to their positions on the chromosome, such as *SiZIP1*, *SiZIP2*.

The basic information of these *ZIP* genes and the proteins they encode was extracted from the genomic annotation files. The information such as protein molecular weight and isoelectric point, transmembrane domains and subcellular localization was obtained respectively from the computer pI/MW tool in the ExPASy database (https://web.expasy.org/compute_pi/), the TMHMM Server 2.0 (http://www.cbs.dtu.dk/services/TMHMM/), and Cell-PLoc 2.0 (http://www.csbio.sjtu.edu.cn/bioinf/plant-multi/).

### 2.2 Duplication, selection pressure and chromosomal distribution of *SiZIP*s

To investigate the expansion pattern of the *ZIP* gene family in foxtail millet, the Multiple Collinearity Scan toolkit (MCscanX) was employed to analyze the duplication relationship of *ZIP* genes in foxtail millet. Meanwhile, for the duplicated gene pairs, the ratio of non-synonymous mutations (Ka) to synonymous mutations (Ks), Ka/Ks, was calculated using KaKs_Calculator 3.0. Finally, the *SiZIP* genes and their duplication relationships on the foxtail millet chromosomes were visualized using the TBtools (version 2.119) (Zhang, 2022; Chen et al., 2023a).

### 2.3 Phylogenetic relationships of ZIP transporters among different species

To clarify the relationships among the members of the *ZIP* gene family in foxtail millet, the protein sequences of ZIP transporters from six species were selected to construct a phylogenetic tree together with SiZIPs, including the monocotyledonous gramineous crops rice, sorghum, maize, barley, and the dicotyledonous plants *Arabidopsis* and potato. Multiple sequence alignment was performed using MEGA 6.06, and the maximum likelihood method was used to construct the phylogenetic tree. The phylogenetic tree was visualized by Interactive Tree of Life (iTOL v6, https://itol.embl.de/).

### 2.4 Gene structure and conserved domain characteristics of *SiZIP*s

To analyze the similarities and differences among different SiZIP transporters, the Motif-based sequence analysis Server (MEME version 5.5.1, http://meme-suite.org) was used to predict the conserved motifs of these SiZIP proteins. The motif width was set from 6-200aa, the number of predicted motifs was 10, and they could be displayed repeatedly. The gene structure information of these *SiZIP* genes was extracted from the genomic annotation file. MEGA 6.06 was used for multiple sequence alignment of all SiZIP sequences and a phylogenetic tree was constructed based on the maximum likelihood method. TBtools was used to visualize protein conserved domains, gene structure, and phylogenetic trees (Sievers et al., 2011). Meanwhile, Escript 3.0 was used to visualize the results of multiple sequence alignment of SiZIP proteins and analyze the relationship between conserved motifs and transmembrane (TM) regions.

### 2.5 *cis*-acting elements and spatiotemporal expression characteristics of *SiZIP*s

To elucidate the sequence characteristics of the promoter regions of *SiZIPs*, we used TBtools to extract the sequences 2000 bp upstream of the start codon of these genes for cis-acting element prediction. All sequences were uploaded to the PlantCare website (version 1.0, http://bioinformatics.psb.ugent.be/webtools/plantcare/html/) (Yang et al., 2023). After the prediction was completed, the cis-acting elements in the promoter regions were counted and classified to study the response patterns of *SiZIPs*.

The spatiotemporal expression profiles of *SiZIPs* in different tissues during the whole growth period of foxtail millet were obtained through the foxtail millet multi-omics website (MDSi, version 1.0, http://foxtail-millet.biocloud.net/home). These datasets included RNA-seq data of various tissues, including roots, stems, leaves, flowers, and grains of the foxtail millet model material ‘*xiaomi*’ from the seedling stage to the mature stage. The expression levels of all genes were standardized by TPM values.

### 2.6 Response of *SiZIP*s to Cd, Fe, K, Na, cold and drought stresses

To investigate the responses of *SiZIPs* to Cd, Fe, and K ions, the genomic sequencing material ‘*xiaomi*’ was selected for seedling experiment and subjected to stresses of Cd, Fe, and K. The detailed steps for seedling planting and Cd treatment at three different concentrations (5 µM, Cd1; 10 µM, Cd2, 30 µM, Cd3) were referred to our previous article (Yang et al., 2023). As for Fe and K stresses, five treatments were set by controlling the concentrations of Fe and K ions in Hoagland’s nutrient solution: normal Fe and K (CK), Fe deficiency (0 µM, LFe), high Fe (600 µM, HFe), low K (0.1 mM, LK) and high K (10 mM, HK). The seedlings and roots were rapidly frozen in liquid nitrogen and sent to PERSONAL GENE TECHNOLOGY Co., Ltd. (Nanjing, China) for transcriptome sequencing. cDNA library construction and transcriptome sequencing were performed on the BGISEQ500 sequencing platform and followed the company’s standard sequencing and analysis procedures (Yang et al., 2023). The sequencing raw data was deposited in the NCBI Sequence Read Archive (SRA) with the accession number PRJNA1146895. Meanwhile, the seedlings and roots after Cd treatments were also used for ionome determination of 9 metal ions including Cd, Fe, Zn, Mn, Cu, Mg, Ca, K, Na and Mo on the Thermo Fisher Scientific iCAP TQ ICPMS/MS platform.

Furthermore, to clarify the expression characteristics of *SiZIPs* under salt stress, cold stress and drought stress, we added gene expression datasets obtained from some public databases and unpublished transcriptomes, including the transcriptomes of foxtail millet seedlings after treatment with 150 mM NaCl for 0.5 h and 2 h (unpublished), the transcriptomes of foxtail millet seedlings after cold stress for 1 h, 3 h and 6 h (SRA accession: PRJNA343268), and the transcriptomes of foxtail millet seedlings during the day/night after long-term drought (unpublished). The gene expression levels under all these different treatments were standardized using TPM values (Transcripts Per Kilobase of exon model per Million mapped reads).

### 2.7 Variation analysis and functional prediction of *SiZIP*s

To explore the sequence variation and expression level variation of *SiZIPs* in natural populations and further evaluate the role of *SiZIPs* in the quality improvement of foxtail millet. We analyzed the previously completed genomic resequencing data of 360 foxtail millet genotypes and the transcriptome data of grains at the filling stage (Li et al., 2022), and obtained the sequence variation information and expression data of *SiZIPs* in 360 genotypes based on manual screening.

Considering the great progress of rice in the study of gene functions and the widespread convergent evolutionary characteristics of gramineous crops, a foxtail millet-rice homology comparison strategy was used to further analyze the functions of *SiZIPs*. The orthologs of *SiZIPs* in rice were detected by MCscanX and the phylogenetic tree. On this basis, we conducted an in-depth and detailed literature survey to search for the reported functions of *OsZIPs*. Additionally, the expression patterns of these genes across different tissues were obtained from the Rice Expression Database (https://rapdb.dna.affrc.go.jp/) (Chen et al., 2023b). By comparing the expression characteristics of *ZIP* genes in foxtail millet and rice, along with the reported functions of *OsZIP* genes, we inferred the potential functions of *SiZIP* genes. In addition, the PPI (protein-protein interaction) networks of SiZIPs were predicted through the STRING database (Search Tool for the Retrieval of Interacting Genes/Proteins, version 12.0, https://cn.string-db.org/).

## 3 Results

### 3.1 Basic characteristics of *SiZIP*s

Through local BLASTP and after eliminating sequences of different transcripts from the same gene as well as those containing incomplete conserved domains, 14, 14, 12, and 12 *ZIP* genes were identified in foxtail millet, sorghum, maize, potato, respectively. Compared with 13 in rice and 15 in *Arabidopsis*, the number of *ZIPs* did not exhibit significant disparities between monocotyledons and dicotyledons, and no conspicuous uneven expansion was discerned among different species.

Based on their locations on the chromosomes, the *SiZIPs* were designated as *SiZIP1* to *SiZIP14* and the detailed information was listed in **Table 1**. Overall, *SiZIPs* are relatively short with lengths ranging from 1062 bp for *SiZIP3* to 2637 bp for *SiZIP8*. Except for *SiZIP8* with 10 introns, the number of introns in other *SiZIPs* varies from 1 to 3. The 14 SiZIP proteins range in size from a minimum of 271 amino acids (aa) to a maximum of 474 aa and the corresponding molecular weights range from a minimum of 28678.23 Da to a maximum of 43731.21 Da. As typical transmembrane transporters, all SiZIPs contain varying numbers of TM regions with the number of TMs ranging from 4 to 9. The subcellular localization results based on Loctree 3 and Cell-PLoc indicated that all SiZIPs were localized on the membrane. In general, SiZIPs exhibited rich diversity in terms of gene structure, protein physicochemical properties, and TM regions.

**Table 1:** The detail information and sequence characterization of 14 putative *ZIP* genes in foxtail millet.

No.	Gene^a^	Locus^b^	Chr^c^	Start	End	Gene Structure		Protein			TM region^f^	Subcellular Localization	Gene duplication^g^
						Length	Intron	Size	MW^d^	pI^e^			
1	SiZIP1	Si2g09630	Chr2	8724488	8727910	1917	2	393	6.08	40147.80	7	membrane.	
2	SiZIP2	Si3g11480	Chr3	7639607	7642733	2045	3	389	6.99	40760.09	6	membrane.	SD1
3	SiZIP3	Si3g20470	Chr3	16071601	16075462	1062	2	353	6.05	36313.70	7	membrane.	TD1
4	SiZIP4	Si3g20480	Chr3	16076742	16080702	1194	2	354	6.04	36824.20	9	membrane.	TD1
5	SiZIP5	Si4g22770	Chr4	34067341	34070180	2106	3	424	6.38	43731.21	6	membrane.	SD1
6	SiZIP6	Si5g46440	Chr5	47855758	47858853	1351	2	348	6.79	37643.84	9	membrane.	
7	SiZIP7	Si6g03490	Chr6	2139910	2143676	2364	2	399	7.82	40921.63	7	membrane.	SD2
8	SiZIP8	Si6g17940	Chr6	29249076	29257615	2637	10	474	6.16	50656.58	7	membrane.	
9	SiZIP9	Si7g20470	Chr7	26735106	26743568	1349	2	359	6.45	37714.10	9	membrane.	
10	SiZIP10	Si7g24380	Chr7	29825058	29827443	1937	1	271	6.92	28678.23	4	membrane.	SD2
11	SiZIP11	Si7g27930	Chr7	32503074	32507011	2555	1	406	6.26	42120.95	8	membrane.	
12	SiZIP12	Si9g14380	Chr9	9666752	9670672	1527	1	377	9.12	39626.58	8	membrane.	TD2
13	SiZIP13	Si9g14390	Chr9	9673208	9674738	1452	1	382	7.7	40200.08	9	membrane.	TD2
14	SiZIP14	Si9g36300	Chr9	43462111	43466102	2223	3	353	6.18	36586.30	9	membrane.	

^a^ Systematic designation of ZIPs in foxtail millet; ^b^ Locus identity number of SiZIPs in foxtail millet genome (cv ‘xiaomi’); ^c^ chromosome;

^d^ Molecular weight (Da); ^e^ isoelectric point; ^f^ The number of transmembrane regions; ^g^ SD, segmental duplication; TD, tandem duplication.

### 3.2 Chromosomal localization, expansion and selective pressure of *SiZIP*s

All 14 *SiZIPs* were unevenly distributed across 7 chromosomes with chromosomes 3, 7, and 9 each having the highest number of 3 genes. Chromosome 6 contains 2 genes and the remaining genes are distributed on chromosomes 2, 4, and 5 (**Supplementary Figure S1**). Overall, *SiZIPs* were mainly located at both ends of the chromosomes, which in line with the distribution characteristics of foxtail millet genes on chromosomes. Gene duplication analysis showed that 8 *SiZIPs* participated in tandem duplication (TD) and segmental duplication (SD) events, forming two TD gene pairs (*SiZIP3* & *SiZIP4*, TD1; *SiZIP12* & *SiZIP13*, TD2) and two SD gene pairs (*SiZIP2* & *SiZIP5*, SD1; *SiZIP7* & *SiZIP10*, SD2). Although the Ka/Ks values of all duplicated gene pairs were less than 1, the TD gene pairs (0.32 and 0.43) had higher Ka/Ks values than the SD gene pairs (0.09 and 0.24). These results indicated that both TD and SD have contributed to the expansion of *ZIP* gene family in foxtail millet.

### 3.3 Phylogenetic relationships of ZIP transporters among different species

**Figure 1:**
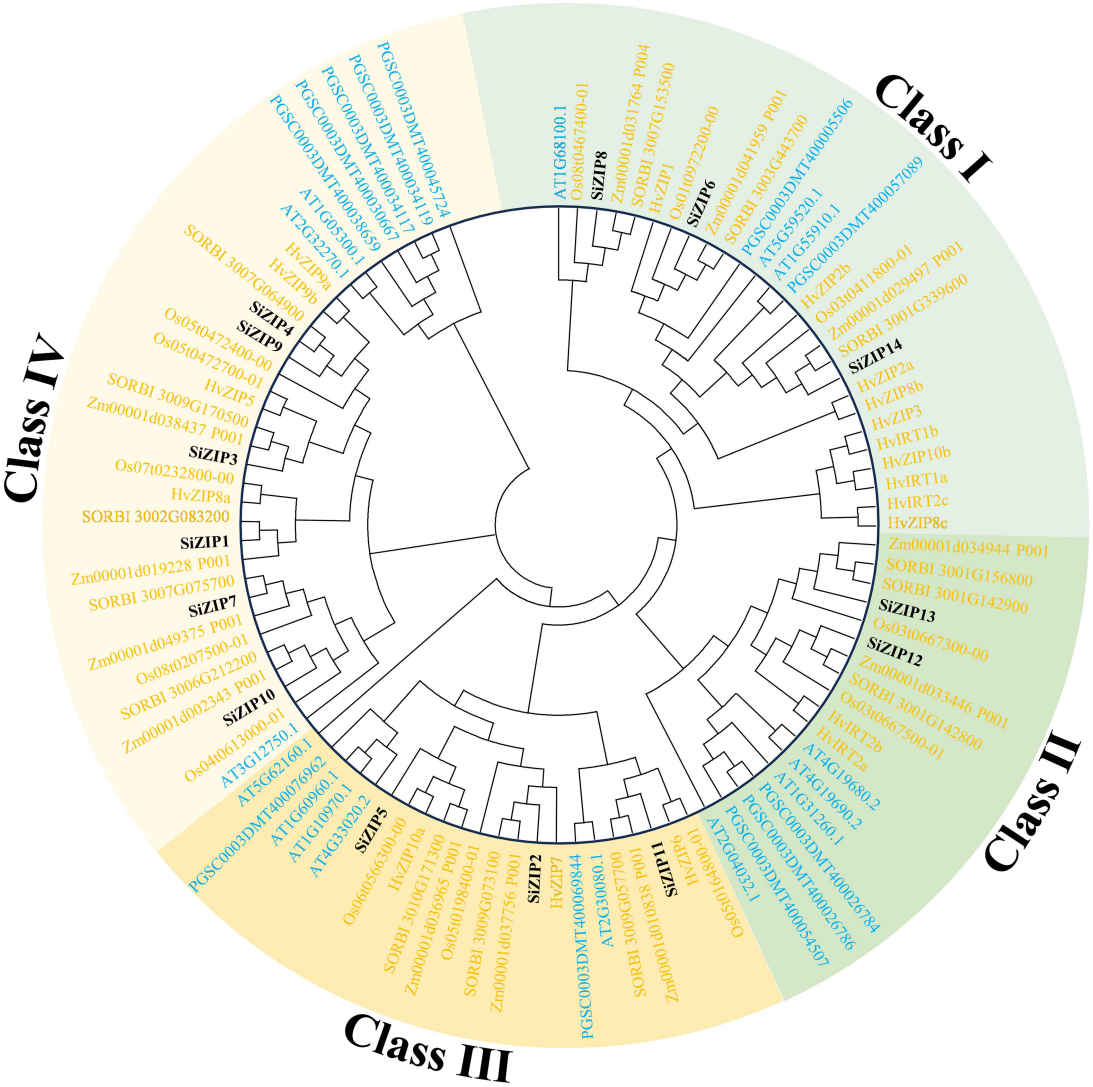
The phylogenetic tree of ZIP transporters in seven plants. The phylogenetic tree is constructed by ZIP proteins of foxtail millet, sorghum, maize, rice, barley, *Arabidopsis* and potato using MEGA 6 with maximum likelihood method. Different clades and ZIPs from monocots and dicots are distinguished by different colors.

To clarify the phylogenetic relationships of the ZIP family in different species, we constructed a phylogenetic tree based on protein sequences for 99 ZIP proteins from seven species (dicots: *Arabidopsis* and potato; monocots: foxtail millet, sorghum, maize, rice, and barley). The results showed that all ZIP proteins were divided into four major clades, Class I-IV, and all clades contained ZIP transporters from dicots and monocots, indicating that the *ZIP* gene family was formed before the differentiation of dicots and monocots (**Figure 1**).

Class I consisted of 27 ZIPs, including 3 SiZIPs of foxtail millet, 3 OsZIPs of rice, 3 ZmZIPs of maize, 3 SbZIPs of sorghum, 10 HvZIPs of barley, 3 AtZIPs and 2 StZIPs of potato, forming three orthologous clusters (OCs, OC1–3) (**Table 2**). Except for the significant expansion of HvZIPs in Class I, ZIPs of other species were relatively conserved in monocots and dicots. Class II contained 18 ZIPs from different species, including 2 SiZIPs, 2 OsZIPs, 2 ZmZIPs, 3 SbZIPs, 2 HvZIPs, 4 AtZIPs and 3 StZIPs. Although the number of ZIPs of different species in Class II did not show a significant difference, ZIPs of monocots and dicots in Class II were located in different branches. Furthermore, Class II included multiple tandem duplicate gene pairs of monocots and dicots, which confirmed that the ZIP genes in Class II were formed by independent duplication after the differentiation of monocots and dicots (OC 4-1 and OC 4-2). Class III comprised 22 ZIP proteins, including 3 SiZIPs, 3 OsZIPs, 3 ZmZIPs, 3 SbZIPs, 3 HvZIPs, 5 AtZIPs and 2 StZIPs, forming an OC (OC5) containing ZIP proteins from seven species and two OCs (OC6-1 and OC6-2) consisting of five monocotyledonous ZIP proteins. Moreover, the tandem duplication of *AtZIPs* in Class III also promotes the expansion of ZIP gene family in *Arabidopsis*. Class IV consisted of 32 ZIPs. Besides including one orthologous gene cluster OC 7 composed of ZIPs of monocots and dicots, it also contained 4 homologous gene clusters (OC 8-1 to OC 8-4) specific to monocots. The remaining ZIPs in *Arabidopsis* and potato were formed after the divergence of monocots and dicots. Overall, ZIP proteins, apart from those of Class I being highly conserved, displayed a certain level of unequal duplication and expansion in monocots and dicots in other classes, forming multiple ZIP clusters unique to monocots.

**Table 2:** Homologous relationships of *ZIP* genes in seven species.

Groups	Cluster^a^	Dicots		M_cluster^b^	Monocots				
		Arabidopsis	Solanum tuberosum		Foxtail millet	Oryza sativa	Zea mays	Sorghum bicolor	Hordeum vulgare
Class I	OC 1	AT1G68100		OC 1-1	SiZIP8	Os08t0467400	Zm00001d031764	SORBI_3007G153500	
	OC 2	AT5G59520	PGSC0003DMT400005506	OC 2-1	SiZIP6	Os01t0972200	Zm00001d041959	SORBI_3003G443700	HvZIP1
	OC 3	AT1G55910	PGSC0003DMT400057089	OC 3-1	SiZIP14	Os03t0411800	Zm00001d029497	SORBI_3001G339600	HvZIP2b
	PC								HvZIP2a, HvZIP8b, HvZIP3, HvZIP3, HvIRT1b, HvZIP10b, HvIRT1a, HvIRT2c, HvZIP8c
Class II	OC 4	**AT4G19680** **AT4G19690** AT1G31260 AT2G04032	**PGSC0003DMT400026784** **PGSC0003DMT400026786** PGSC0003DMT400054507	OC 4-1	**SiZIP13**	**Os03t0667300**	Zm00001d034944	SORBI_3001G156800, **SORBI_3001G142900**	
				OC 4-2	**SiZIP12**	**Os03t0667500**	Zm00001d033446	**SORBI_3001G142800**	HvIRT2b, HvIRT2a
Class III	OC 5	AT2G30080	PGSC0003DMT400069844	OC 5-1	SiZIP11	Os05t0164800	Zm00001d010838	SORBI_3009G057700	HvZIP6
	OC 6	AT1G10970 **AT1G60960** **AT1G60970**	PGSC0003DMT400076962	OC 6-1	SiZIP2	Os05t0198400	Zm00001d037756	SORBI_3009G073100	HvZIP7
				OC 6-2	SiZIP5	Os06t0566300	Zm00001d036965	SORBI_3010G171300	HvZIP10a
Class IV	OC 7	AT3G12750		OC 7-1	SiZIP10	Os04t0613000	Zm00001d002343	SORBI_3006G212200	
	OC 8			OC 8-1	SiZIP7	Os08t0207500	Zm00001d049375	SORBI_3007G075700	
				OC 8-2	SiZIP1	Os07t0232800	Zm00001d019228	SORBI_3002G083200	HvZIP8a
				OC 8-3	SiZIP3	**Os05t0472700**	Zm00001d038437	SORBI_3009G170500	HvZIP5
				OC 8-4	SiZIP4, SiZIP9	**Os05t0472400**		SORBI_3007G064900	HvZIP9a, HvZIP9b
PC	AT1G05300 AT2G32270	PGSC0003DMT400045724 **PGSC0003DMT400034119** **PGSC0003DMT400034117** PGSC0003DMT400030667 PGSC0003DMT400038659						

^a^ OC, Orthologous cluster; PC, Paralogous cluster; ^b^ M_cluster represents the unique gene cluster of monocots. The tandem duplicated gene pairs are bold.

### 3.4 Gene structure and conserved domain characteristics of *SiZIP*s

**Figure 2:**
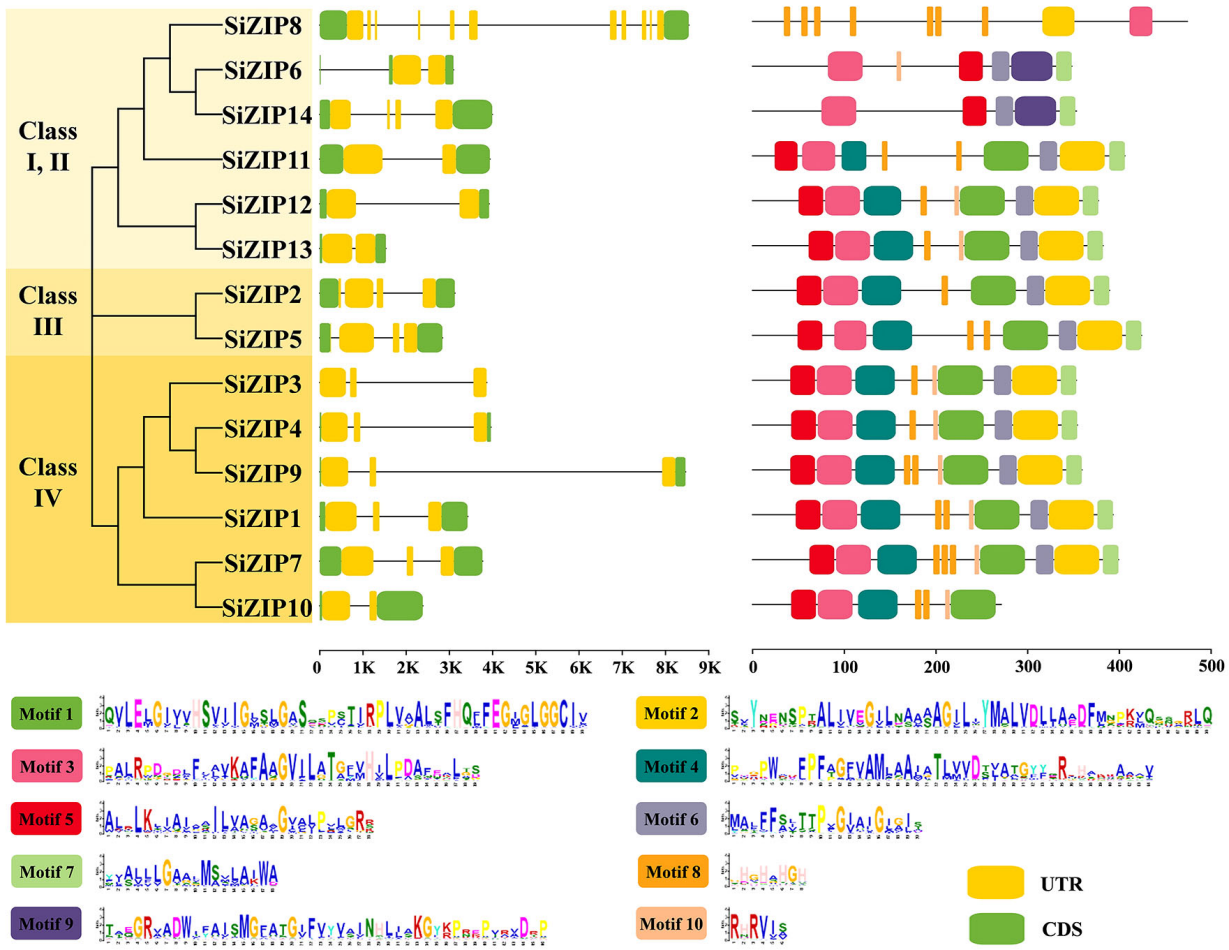
Gene structure and conserved motifs of SiZIPs in foxtail millet. Different classes are distinguished by different colors, and the gene structure and conserved motifs are derived from genome annotation file and MEME prediction, respectively.

To compare the characteristics of different *SiZIP* genes, we analyzed their gene structures and protein conserved domains (**Figure 2**). Overall, *SiZIP* genes within the same class exhibit relative conservation in gene structure. For instance, *SiZIP3*, *SiZIP4*, *SiZIP9* and *SiZIP1* in Class IV contained two introns, while *SiZIP2* and *SiZIP5* in Class III had three introns. However, some *SiZIP* genes also displayed some variations, such as *SiZIP6* and *SiZIP14*. Furthermore, the two genes of paralogous gene pairs also showed characteristics of the coexistence of similarity in conservation and diversity in gene structure. For example, the gene structures of *SiZIP2* and *SiZIP5* (SD1) were similar, while *SiZIP7* and *SiZIP10* (SD2) showed differences. In terms of the conserved domains, some motifs were conserved in all SiZIP transporters, such as Motif 5, Motif 3 and Motif 6, while some motifs were specific in some SiZIPs. For instance, Motif 9 only exists in SiZIP6 and SiZIP14. Regarding paralogous gene pairs, the conserved domains of SiZIP12 and SiZIP13 (TD2) were basically the same, while SiZIP10 lacked three motifs compared to SiZIP7. In general, *SiZIPs* maintained a certain level of conservation in both gene structure and protein conserved domains, while also generating novel variations, especially among paralogous gene pairs.

**Figure 3:**
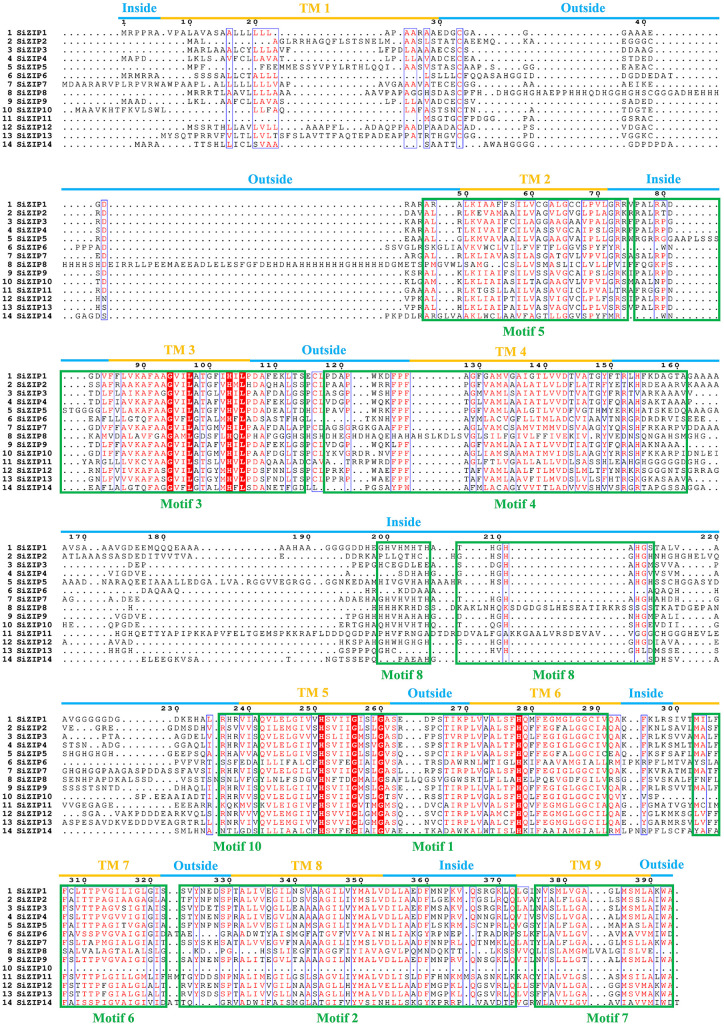
The multiple-sequence alignment of SiZIPs in foxtail millet. Clustal Omega and Escript 3.0 are used to construct the multiple sequence alignment and visualization respectively. The transmembrane (TM) regions and conserved motifs are labeled by blue/orange lines and green boxes, respectively.

The relationship between the TM regions and conserved motifs of these SiZIP proteins was further compared (**Figure 3**). The results showed that a large number of conserved motifs highly coincided with the TM regions, such as Motif 5 - TM 2, Motif 4 - TM 4, Motif 6 - TM 7 and Motif 7 - TM 9. Some other motifs contained TM regions and some intra- and extra-membrane structures. For example, Motif 3 contained TM 3 and the intra-membrane part, and Motif 1 contained TM regions TM 5 and TM 6 and the extra-membrane part in the middle. The absence of conserved motifs in SiZIP proteins was directly related to the number of their TM regions.

### 3.5 *Cis*-acting elements and spatiotemporal expression characteristic of *SiZIP*s

**Figure 4:**
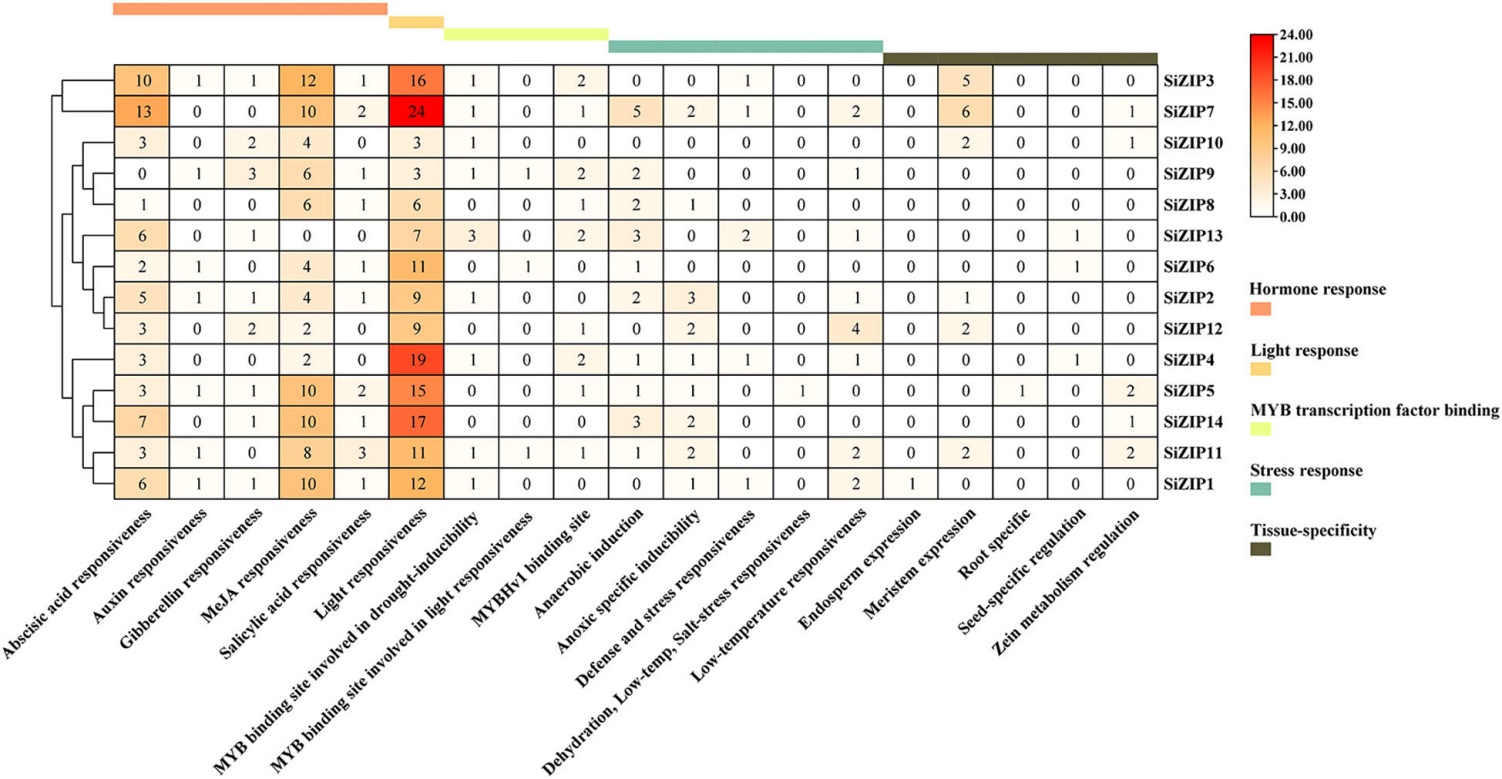
The quantity distribution of different *cis*-acting elements in the promoter regions of *SiZIPs*. All cis-acting are predicted online by PlantCare and manually classified. The numbers in the figure represent the quantity of cis-acting elements in the promoter region of the *SiZIP* genes.

We predicted and analyzed the *cis*-acting elements in the promoter regions of all *SiZIP* genes (**Figure 4**). The promoter regions of *SiZIPs* mainly contained five types of *cis*-acting elements: hormone response, light response, MYB transcription factor binding, stress response and tissue-specific expression. The hormone response elements included abscisic acid response, auxin response, gibberellin response, MeJA response and salicylic acid response elements. The abscisic acid response elements and MeJA response elements were the most abundant, followed by gibberellin response elements and salicylic acid response elements, indicating that these SiZIP genes are regulated by plant growth regulators to varying degrees. Multiple MYB transcription factor binding sites were predicted in the promoter regions of *SiZIPs* such as *SiZIP13* and *SiZIP9*, suggesting that these genes may be regulated by MYB transcription factors. In addition, the stress response elements included various stresses such as anaerobic induction, drought, low temperature, and salt, confirming that these genes might be involved in the response to various osmotic stresses. Finally, The presence of tissue-specific expression elements, including endosperm-, root-, meristem-, and seed-specific expression elements, was likely a significant factor contributing to the diversity of tissue-specific expression in *SiZIPs*.

**Figure 5:**
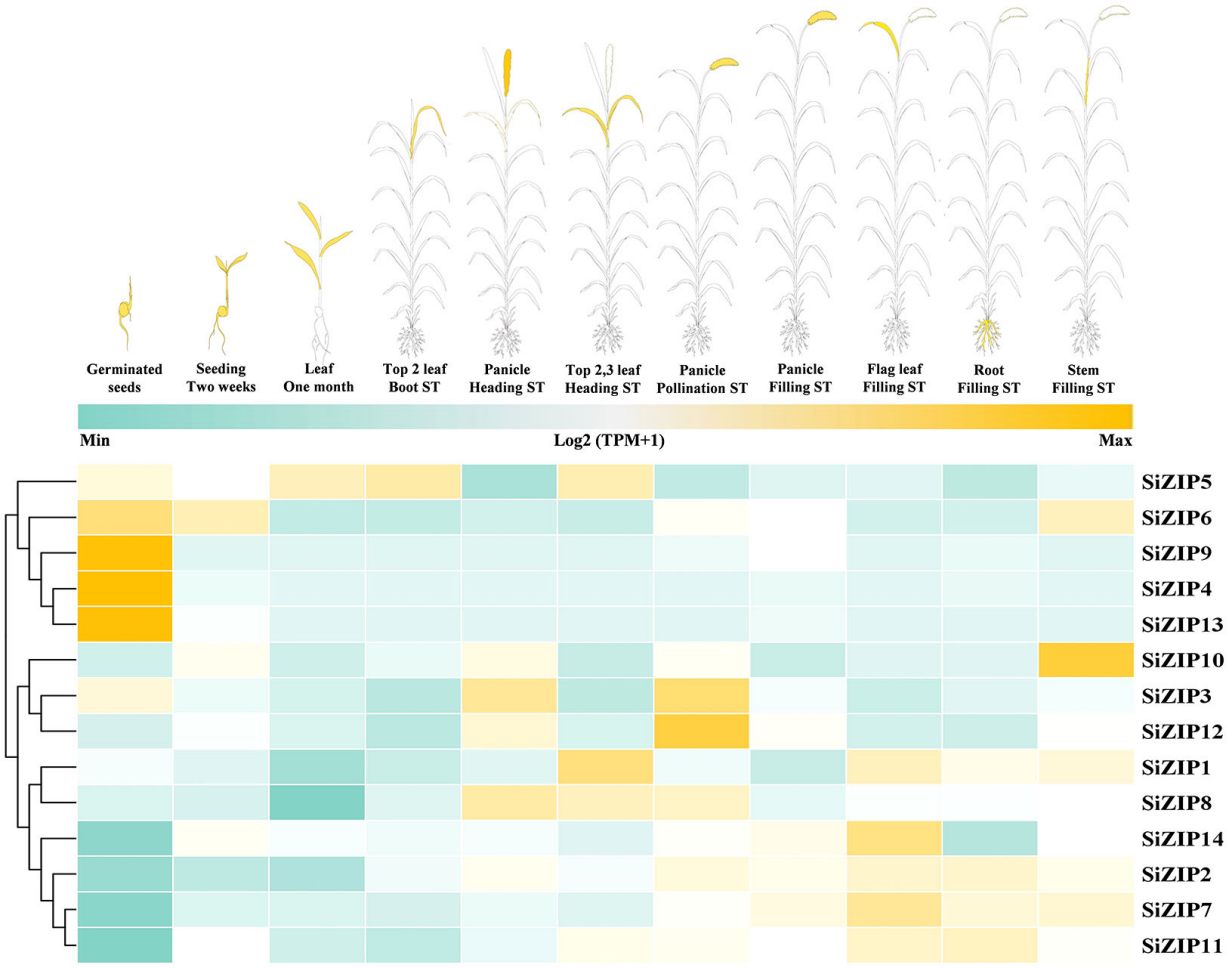
The spatiotemporal expression patterns of *SiZIPs* in multiple tissues during whole growth period in foxtail millet. The expression matrices (TPM values) are obtained from foxtail millet multi-omics database (MDSi). ST represents stage.

*SiZIPs* exhibited diverse expression patterns throughout the whole growth period of foxtail millet, demonstrating clear temporal and spatial specificity (**Figure 5**; **Supplementary Figure S2**). Overall, the expression patterns of *SiZIPs* could be broadly classified into three categories. The first category was highly expressed during the germination and the subsequent seedling growth, with *SiZIP9*, *SiZIP4* and *SiZIP13* specifically expressed in germinating seeds and *SiZIP6* expressed in both germinating seeds and seedlings. The second category was primarily expressed in panicles and leaves during the heading and flowering stages, such as *SiZIP8* and *SiZIP3*. The third category was expressed in various tissues during the grain-filling stage, such as *SiZIP14* and *SiZIP7*. Regarding tissue-specific expression, *SiZIP10* was specifically expressed in stems, *SiZIP5* and *SiZIP1* were primarily expressed in leaves, *SiZIP12* was expressed in panicles during multiple stages, and *SiZIP2*, *SiZIP7* and *SiZIP11* were highly expressed in roots. Furthermore, paralogous gene pairs also exhibited differences in expression patterns. For example, in SD1, *SiZIP2* was expressed in multiple tissues including roots, stems and leaves, while its paralog *SiZIP5* was primarily expressed in leaves. In TD1, *SiZIP3* was primarily expressed in panicles, while its paralog *SiZIP4* was specifically expressed in germinating seeds. The diverse temporal and spatial expression patterns exhibited by different members of the *SiZIP* gene family, along with the emergence of novel expression patterns in newly duplicated genes, contributed significantly to the functional diversification of *SiZIPs*.

### 3.6 Response of *SiZIP*s to different metal ions and abiotic stresses

**Figure 6:**
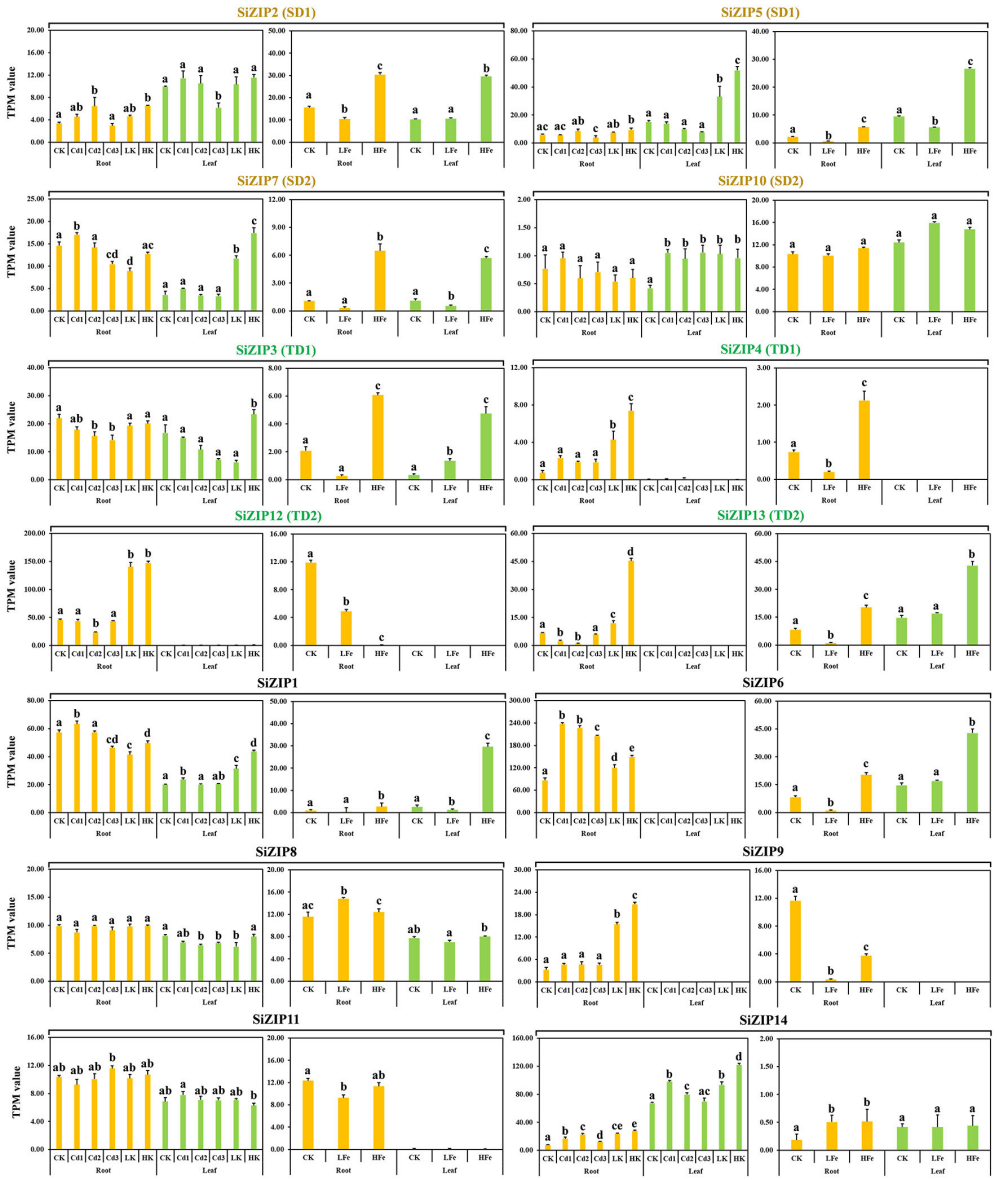
The expression patterns of 14 *SiZIPs* under Cd, Fe and K treatments. The Y-axis represents the TPM value based on RNA-seq. Bars represent the mean values of three replicates ± standard deviation (SD). Cd1, Cd2, Cd3, LK, HK, LFe and HFe represent 5 µM Cd treatment, 10 µM Cd treatment, 30 µM Cd treatment, 0.1 mM K treatment, 10 mM K treatment, 0 uM Fe treatment and 600 µM Fe treatment, respectively. Significant differences (P < 0.05) between groups are marked with different letters.

*SiZIPs* exhibited distinct expression patterns in different tissues in response to varying concentrations of Cd, Fe, and K stress (**Figure 6**). Except for *SiZIP10*, *SiZIP8* and *SiZIP11*, which did not respond to Cd, Fe and K treatments, the expression of the remaining 11 *SiZIP*s was inhibited or induced by different metal ions. As important Zn and Fe transporters, 8 genes (*SiZIP2*, *SiZIP5*, *SiZIP7*, *SiZIP3*, *SiZIP4*, *SiZIP13*, *SiZIP1* and *SiZIP6*) showed significantly upregulated expression under high Fe treatment. Interestingly, some *SiZIPs* were induced to be expressed by both high Fe and high K simultaneously. While *SiZIP12* and *SiZIP9* were significantly suppressed under different concentrations of Fe treatment but significantly upregulated under different K treatments. In addition, the expression patterns of duplicated genes under different metal treatments were also diverse. For example, *SiZIP7* in SD2 was significantly induced under high Fe and high K treatments, while *SiZIP10* did not respond to any metal ions. In TD1, *SiZIP3* was expressed in both roots and leaves and was not significantly induced by K, while *SiZIP4* was only expressed in roots and was significantly induced and upregulated by K. Overall, the *SiZIP* genes exhibited diverse responses and expression patterns under different ionic stresses.

To clarify the correlation between *SiZIP* gene expression and metal ion transport, we measured the contents of 10 metal ions (Cd, K, Fe, Ca, Na, Mg, Mn, Cu, Zn and Mo) in the roots and leaves of foxtail millet seedlings after Cd stresses (**Supplementary Figure S3A**). Among these ions, besides Cd, Fe (318.9-657.6 mg/kg), Ca (345.0-1880.4 mg/kg), K (2249.8-14230.1 mg/kg) and Na (31.5-193.1 mg/kg) all showed certain differences between Cd treatments and CK. The correlation analysis revealed significant correlations between the expression levels of different *SiZIPs* in various tissues and the contents of these metal ions. Specifically, *SiZIP4*, *SiZIP6*, *SiZIP7* and *SiZIP9* showed significant positive and negative with Fe (at least 0.57) and K (0.81-0.90) content, respectively, which directly correlated with their induced expression under Fe and K stresses (**Supplementary Figure S3B**). *SiZIP12*, which was induced by K, exhibited a significant negative correlation (0.85) with K content. Although the dataset used for correlation analysis was not large, it suggested to a certain extent that *SiZIPs* were closely related to the transport and accumulation of different metal ions in foxtail millet, especially Fe.

**Figure 7:**
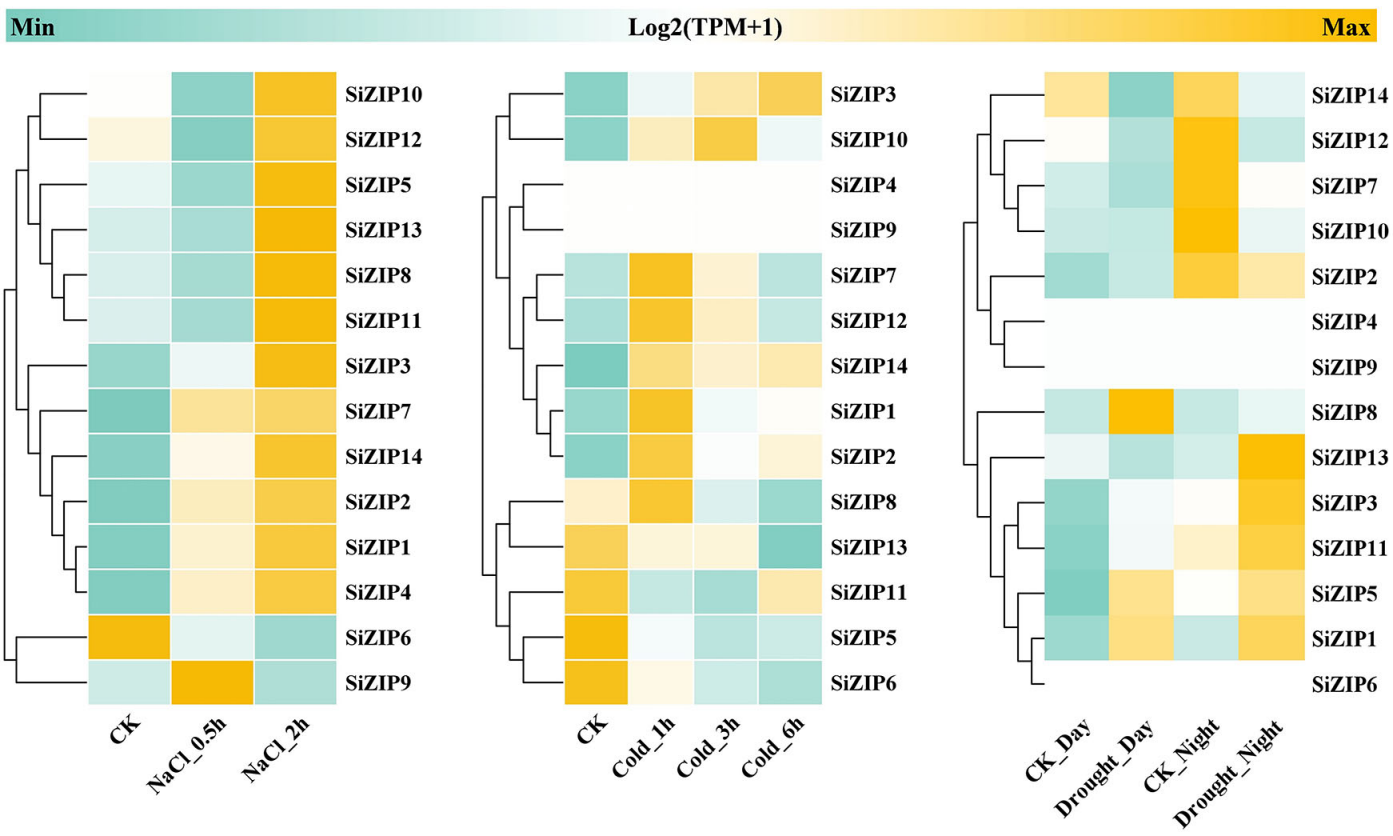
The heatmaps of *SiZIP* expression under salt stress, cold stress and drought stress. From left to right are heatmaps of expression levels of *SiZIP*s in foxtail millet seedlings under salt stress (150 mM NaCl), cold stress (4°C) and drought stress. All expression data are obtained from RNA-seq and the gene expression levels are standardized using TPM values (Transcripts Per Kilobase of exon model per Million mapped reads). The log_2_ (TPM+1) values are used to display the expression levels.

**Figure 8:**
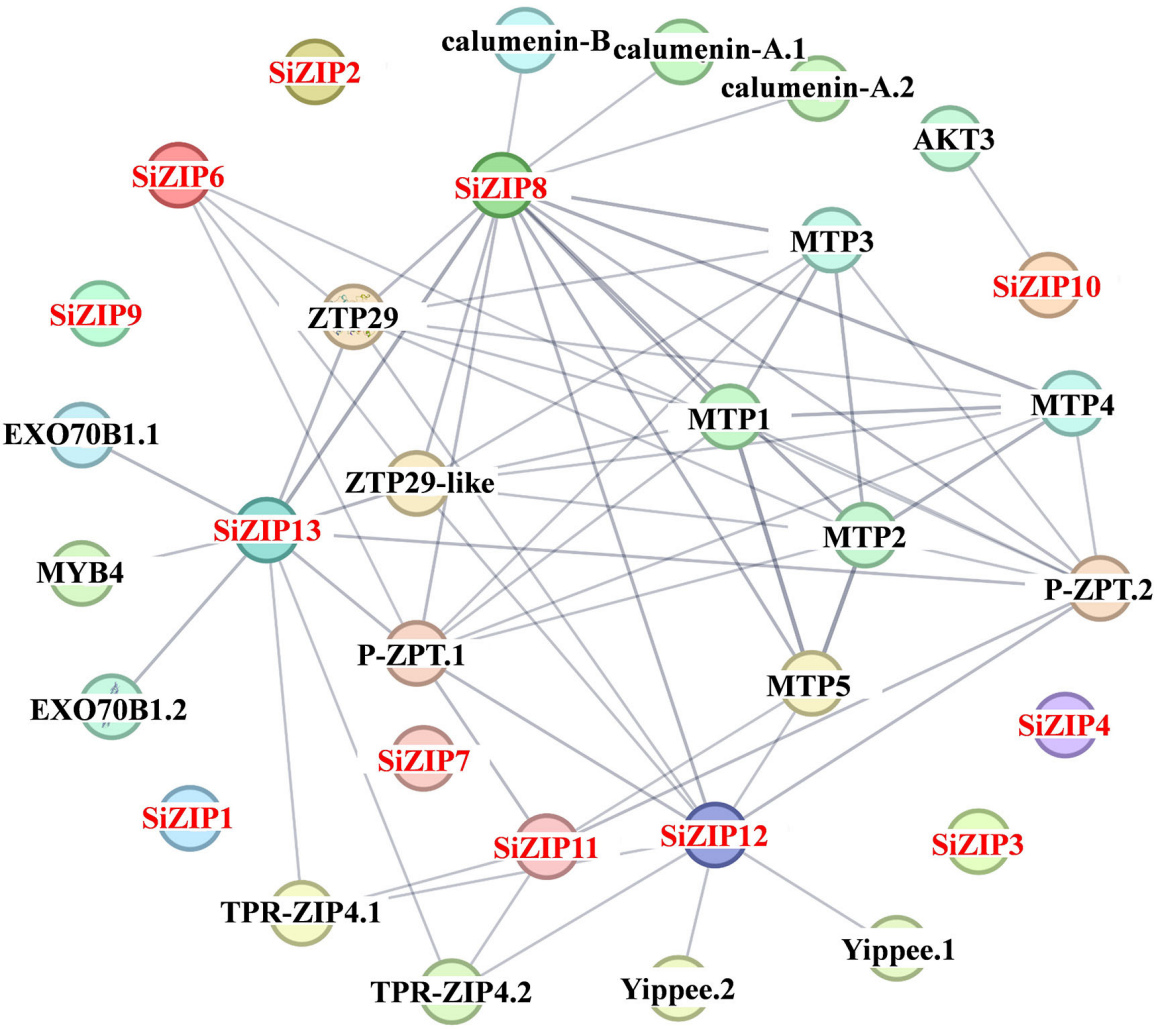
The protein-protein interaction networks of SiZIPs with other proteins in foxtail millet. The protein-protein interaction networks of SiZIPs were predicted by the STRING database (Search Tool for the Retrieval of Interacting Genes/Proteins, https://cn.string-db.org/). The thickness of the line represents the degree of interaction between different proteins. MTP: metal tolerance protein; AKT3: potassium channel AKT3; EXO70B1: exocyst subunit Exo70 family protein; ZTP29/ZTP29-like: zinc transporter ZTP/ZTP29-like protein; TPR-ZIP4: TPR repeat-containing protein ZIP4; P-ZRT: putative zinc transporter; Yippee: yippee family putative zinc-binding protein.

In addition to analyzing the response of *SiZIPs* to metal ions, we have also analyzed their response to various abiotic stresses (**Figure 7**). Except for *SiZIP6*, all other *SiZIP*s were induced to up-regulate their expression under salt stress. The weak response of *SiZIP6* to Na and K and its strong up-regulation under Fe and Cd stresses suggested that it might be involved in the transport of Fe and Cd in foxtail millet simultaneously. Multiple *SiZIPs*, including *SiZIP3*, *SiZIP10*, *SiZIP7*, *SiZIP14* and *SiZIP8*, were induced to up-regulate their expression under cold stress, especially in the early stage (1 h). *SiZIP13*, *SiZIP3*, *SiZIP11*, *SiZIP5* and *SiZIP1* were induced to up-regulate their expression after drought stress. The results indicated that these *SiZIPs* might enhance the tolerance of seedlings to abiotic stresses by altering the transport and distribution of metal ions in foxtail millet and regulating the osmotic potential inside and outside the membrane under abiotic stresses. Furthermore, The PPI results showed that except for a certain degree of interaction between SiZIP13, SiZIP8, SiZIP11, and SiZIP12, the functions of other SiZIP proteins were relatively independent (**Figure 8**). However, interactions between SiZIPs and MTPs (Metal Tolerance Protein), potassium channel protein, EXO (Exocyst subunit Exo70 family protein), calumenin protein, other Zn transporters, and MYB transcription factors were discovered. For example, SiZIP8 interacted with ZTP29, MTP1 and calumenin-B, SiZIP13 interacted with MYB4 and EXO70B1. This suggested that SiZIPs might play a role in metal transport in conjunction with other metal transporters and exosome related proteins, and these transporters were also regulated by transcription factors.

### 3.7 Sequence and expression variation of *SiZIPs* in 360 foxtail millet genotypes

A total of 1718 genetic variation loci were identified among 360 foxtail millet genotypes for 14 *SiZIP* genes, including 1525 SNPs and 193 Indels (**Table 3**). Among them, 54 variations were non-synonymous SNPs, and 235 variations were located in the promoter regions. To explore the expression variations of *SiZIPs* in different genotypes, we investigated the expression levels of *SiZIPs* in grains during the filling stage across 360 genotypes (**Supplementary Figure S4**) (Li et al., 2022). The expression levels of *SiZIP* genes showed significant differences among 360 accessions. For example, *SiZIP14* was expressed at a low level (TPM < 25) in most foxtail millet genotypes, but up to a high level in a few genotypes (TPM = 80). The results confirmed the extensive sequence and expression variations of *SiZIPs* in the natural foxtail millet population, suggesting their genetic selection potential in foxtail millet breeding.

**Table 3:** Genomic variation of 14 *SiZIPs* in 360 foxtail millet accessions.

Gene	Type of variations	No.	Exonic (synonymous)	Exonic (nonsynonymous)	Intronic	5’-UTR	3’-UTR	Upstream	Downstream
SiZIP1	SNP	70	3	4	33	0	7	14	9
	Indel	24	0	0	14	0	4	2	4
	Total	94	3	4	47	0	11	16	13
SiZIP2	SNP	80	1	0	12	3	7	29	28
	Indel	5	0	0	0	0	1	2	2
	Total	85	1	0	12	3	8	31	30
SiZIP3	SNP	182	5	5	142	0	0	9	21
	Indel	18	0	1	8	0	0	1	8
	Total	200	5	6	150	0	0	10	29
SiZIP4	SNP	125	3	2	101	1	1	10	7
	Indel	16	0	0	12	0	0	2	2
	Total	141	3	2	113	1	1	12	9
SiZIP5	SNP	45	4	7	6	3	1	19	5
	Indel	11	0	1(stopgain)	1	3	1	5	0
	Total	56	4	8	7	6	2	24	5
SiZIP6	SNP	124	10	4	25	1	2	4	78
	Indel	14	0	0	7	4	1	1	1
	Total	138	10	4	32	5	3	5	79
SiZIP7	SNP	87	9	1	10	3	5	35	24
	Indel	15	0	1	4	1	1	6	2
	Total	102	9	2	14	4	6	41	26
SiZIP8	SNP	109	4	4	47	4	17	31	2
	Indel	15	0	0	7	0	5	3	0
	Total	124	4	4	54	4	22	34	2
SiZIP9	SNP	352	4	1	341	0	0	3	3
	Indel	20	0	0	17	0	0	2	1
	Total	372	4	1	358	0	0	5	4
SiZIP10	SNP	0	0	0	0	0	0	0	0
	Indel	0	0	0	0	0	0	0	0
	Total	0	0	0	0	0	0	0	0
SiZIP11	SNP	61	2	9	0	2	35	8	5
	Indel	4	0	0	0	1	3	0	0
	Total	65	2	9	0	3	38	8	5
SiZIP12	SNP	119	7	3	61	4	3	1	40
	Indel	30	0	0	19	0	3	0	8
	Total	149	7	3	80	4	6	1	48
SiZIP13	SNP	96	7	6	1	8	0	30	44
	Indel	11	0	0	0	0	0	6	5
	Total	107	7	6	1	8	0	36	49
SiZIP14	SNP	75	3	5	10	10	3	11	33
	Indel	10	0	0	3	2	0	1	4
	Total	85	3	5	13	12	3	12	37
SiZIPs	SNP	1525	62	54	789	39	81	204	299
	Indel	193	0	0	92	11	19	31	37
	Total	1718	62	54	881	50	100	235	336

### 3.8 Functional prediction of *SiZIP*s based on homologous comparison and expression characteristics

Extensive studies have confirmed the existence of convergent selection among gramineae crops (Chen et al., 2022). Based on the analysis of orthologous relationships and expression characteristics between different species, several important yield-related genes have been discovered in foxtail millet and wheat (Yang et al., 2021a; Liu et al., 2022). Rice, as a model crop in the family of gramineae, has undergone intensive research on the functions of multiple *OsZIPs*, while *SiZIPs* have not been reported yet. Therefore, we preliminarily predicted the functions of *SiZIPs* through literature investigation and comparison of the expression patterns of orthologous *ZIPs* in foxtail millet and rice (**Table 4**). Out of the 14 *SiZIP* genes, 12 orthologs have been identified in rice, and 8 of them exhibited similar expression patterns in foxtail millet and rice, such as *SiZIP10*, *SiZIP3* and *SiZIP2*. *SiZIP10* was specifically highly expressed in the stems of foxtail millet, while its ortholog *OsZIP3* in rice is reported to be responsible for Zn unloading and distribution in the enlarged vascular bundle xylem. *SiZIP3* was specifically highly expressed in the panicles of foxtail millet at multiple stages, and its ortholog *OsZIP5* was mainly expressed in the panicles and participates in Zn homeostasis. *SiZIP2* was highly expressed in the roots, stems, and panicles of foxtail millet, and its ortholog *OsZIP7* has been proven to be highly expressed in the same tissues in rice, responsible for the loading of Zn/Cd in the root xylem and their transfer from the stem node vascular bundles to the grains. The highly conserved expression patterns of *ZIP* genes in foxtail millet and rice suggested that the functions of these genes in gramineae, at least in foxtail millet and rice, were relatively conserved. In addition to sharing similar tissue expression patterns with their orthologs in rice, some *SiZIP* genes also expressed in other tissues. For example, *SiZIP12*, besides highly expressing in the roots for Zn/Fe absorption and transport similar to its ortholog *OsIRT1*, also exhibited high expression in the panicles, suggesting that it may have evolved new functions in foxtail millet while maintaining its essential roles. Moreover, apart from conserved and partially conserved genes, there were a few *SiZIP* genes that exhibited completely different expression patterns compared to their orthologs in rice. For instance, *SiZIP5* was specifically highly expressed in the leaves of foxtail millet throughout all developmental stages, whereas its ortholog *OsZIP12* was primarily expressed in the grains. It suggested that the functions of ZIP12 may differ in foxtail millet and rice.

**Table 4:** Functional prediction of *ZIPs* in foxtail millet based on homology relationship, expression characteristics and previous studies.

ZIPs in foxtail millet	Expression characteristics in foxtail millet^a^	Orthologs in Oryza	Expression characteristics in rice	Function of OsZIPs in previous study	Orthologs and their functions in *Arabidopsis*	Complementation of yeast metaluptake mutants (y/n)	Possible biological processes of SiZIPs
SiZIP1we	**(1) HS-Leaf**, **FS-Root**, FS-Stem; (2) Up-regulated expression in leaf induced by LK, HK and HFe	OsZIP8 (Os07g0232800) (Lee et al., 2010b)	(1) Main-expressed in **Leaf**, **Root**, Inflorescence, Seed; (2) Up-regulated expression in root and shoot induced by Low-Zn	(1) Zn uptake in root; (2) Regulate the distribution of Zn in different tissues	NA	**OsZIP8**: △zrt1△zrt2 (Y), △fet3△fet4 (N) (Zheng et al., 2018)	**Conserved gene function in Poaceae**; Zn uptake in root and distribution of Zn in different tissues
SiZIP2	**(1) FS-Root**, **HS, PS, FS-Panicle**, **FS-Stem**; (2) Up-regulated expression in root and leaf induced by LCd, MCd, LK, HK and HFe	OsZIP7 (Os05g0198400) (Yang et al., 2009; Tan et al., 2019)	(1) Main-expressed in **Root**, **Inflorescence**, **Seed, parenchyma cells of vascular bundles in roots and nodes**; (2) Up-regulated expression in root induced by Low-Fe	(1) Zn/Cd uptake in root; (2) Long-distance transportation from root/basal node to leaf/panicle/upper node	AtZIP4(AT1G10970) AtZIP10(AT1G31260) AtZIP9(AT4G33020)	**OsZIP7**: △fet3△fet4 (Y), △zrt1△zrt2 (N) **AtZIP4**: △zrt1△zrt2 (N), △fet3△fet4 (N), △ctr1 (Y) **AtZIP10**: △zrt1△zrt2 (Y), △fet3△fet4 (N), △ctr1△ctr3 (N), △smf1 (N) **AtZIP9**: △zrt1△zrt2 (N), △fet3△fet4 (N), △ctr1△ctr3 (N), △smf1 (Y) (Zheng et al., 2018)	**Conserved gene function in Poaceae**; Zn/Cd uptake in root and Long-distance transportation from root/basal node to leaf/panicle/upper node
SiZIP3	(1)**HS,PS-Panicle**; (2)Up-regulated expression in root and leaf induced by High-Fe	OsZIP5 (Os05g0472700) (Tan et al., 2020)	(1)Main-expressed in **Inflorescence/Panicle**; (2) Up-regulated expression in root induced by Low-Zn and High-Cd	(1)Zn/Cd uptake in root; (2)Participate in the regulation of Zn homeostasis; (3) Its functionality is partially redundant with *OsZIP9*	NA	**OsZIP5**: △zrt1△zrt2 (Y) (Zheng et al., 2018)	**Conserved gene function in Poaceae**; Zn/Cd uptake in root, Participate in the regulation of Zn homeostasis and may play an important role in grain Zn accumulation
SiZIP4	(1)**Germination**, **Root**; (2)Up-regulated expression in root induced by Low-Cd, Moderate-Cd, High-Cd, Low-K, High-K, and High-Fe	OsZIP9 (Os05g0472400) (Tan et al., 2020; Huang et al., 2020)	(1)Main-expressed in **root (cortex cell)**, seed; (2) Expression in root induced by Low-Zn and inhibited by High-Cd	(1)Zn uptake in root under Zn limitation; (2)Participate in the regulation of Zn homeostasis;	NA	NA	**Incomplete conservation of gene function**; Besides regulating Zn homeostasis, may play an important role in the germination of foxtail millet
SiZIP5	(1)SS,BS,HS-Leaf; (2)Up-regulated expression in root induced by Low-K, High-K and High-Fe	OsZIP12; OsZIP10 (Os06g0566300) (Zhan et al., 2022)	(1)Main-expressed in seed; (2) Expression in root induced by Low-Zn and High-Cd	Affect the accumulation of Zn and Fe in grains	AtZIP4 (AT1G10970) AtZIP10 (AT1G31260) AtZIP9 (AT4G33020)	**OsZIP7**: △fet3△fet4 (Y), △zrt1△zrt2 (N) **AtZIP4**: △zrt1△zrt2 (N), △fet3△fet4 (N), △ctr1 (Y) **AtZIP10**: △zrt1△zrt2 (Y), △fet3△fet4 (N), △ctr1△ctr3 (N), △smf1 (N) **AtZIP9**: △zrt1△zrt2 (N), △fet3△fet4 (N), △ctr1△ctr3 (N), △smf1 (Y) (Zheng et al., 2018)	**Different function in rice and foxtail millet**; Besides regulating Zn homeostasis, may participate in the distribution of Zn in different tissues
SiZIP6	(1)**Germination**, **Seedling**, **FS-Root**; (2)Up-regulated expression in root induced by Low-Cd, Moderate-Cd, High-Cd, Low-K, High-K, and High-Fe	OsZIP1 (Os01g0972200) (Liu et al., 2019)	(1)Main-expressed in **seedling root**; (2) Up-regulated expression induced by High-Fe, High-Cu and High-Cd	(1) Zn uptake in root; (2) as a metal efflux transporter limiting excess Zn, Cu and Cd accumulation	AtZIP2(AT5G59520) (involve in the uptake of Mn/Zn in Roots and the transport of Mn/Zn to xylem parenchyma)	**OsZIP1**: △fet3△fet4 (N), △zrt1△zrt2 (Y), △smf1 (Y) **AtZIP2**: △zrt1△zrt2 (Y), △fet3△fet4 (N), △ctr1△ctr3 (N), △smf1 (Y), △ctr1(Y) (Zheng et al., 2018)	**Conserved gene function in Poaceae**; Zn/Cd uptake in root and as a metal efflux transporter limiting excess Zn, Cu and Cd accumulation
SiZIP7	(1)**FS-Stem, Peduncle**; (2)Up-regulated expression in root induced by Low-Cd and High-Fe, Up-regulated expression in leaf induced by Low-K, High-K and High-Fe	OsZIP4 (Os08g0207500) (Mu et al., 2021)	(1)Main-expressed in **phloem of node and axillary meristem**; (2) Up-regulated expression induced by Low-Zn	Regulate Zn transport in the vascular system and preferentially distributed to tillering buds and panicle	NA	**OsZIP4**: △zrt1△zrt2 (Y), △fet1△fet3△fet4 (N) (Zheng et al., 2018)	**Conserved gene function in Poaceae**; Zn/Cd uptake in root and as a metal efflux transporter limiting excess Zn, Cu and Cd accumulation
SiZIP8	(1)Germination, Seedling, HS-Leaf, Panicle, PS-Panicle, FS-Root, Leaf, Panicle	OsZIP14 (Os08g0467400)	NA	NA	AtIAR1 (AT1G68100) (Regulating auxin metabolism through Zn transport) (Gate et al., 2024)	NA	may be involved in the regulation of auxin metabolism through Zn transport
SiZIP9	(1)**Germination**; (2)Up-regulated expression in root induced by Low-K and High-K, inhibited by Low-Fe and High-Fe	OsZIP9 (Os05g0472400) (Tan et al., 2020; Huang et al., 2020)	(1)Main-expressed in **seedling root (cortex cell)**, seed; (2) Expression in root induced by Low-Zn and inhibited by High-Cd	(1)Zn uptake in root under Zn limitation; (2)Participate in the regulation of Zn homeostasis;	NA	NA	**Incomplete conservation of gene function**; Besides regulating Zn homeostasis, may play an important role in the germination of foxtail millet
SiZIP10	(1)**FS-Stem**	OsZIP3 (Os04g0613000) (Sasaki et al., 2015)	(1)Main-expressed in **Basal stem**; (2) Expression is not induced by Low-Cu, Low-Fe, Low-Zn and Low-Mn	Responsible for the unloading of Zn from the xylem of the expanded vascular bundle and the preferential distribution of Zn to developing tissues	AtZIP1 (At3g12750)	**OsZIP3**: △fet3△fet4 (N), △zrt1△zrt2 (Y), △smf1 (Y) **AtZIP1**: △zrt1△zrt2 (Y), △fet3△fet4 (N), △ctr1△ctr3 (N), △smf1 (Y) (Zheng et al., 2018)	**Conserved gene function in Poaceae**; Responsible for the unloading of Zn from the xylem of the expanded vascular bundle and the preferential distribution of Zn to developing tissues
SiZIP11	(1)**HS-Leaf**, **PS-Panicle**, **PS-Leaf**, **Stem**, **Leaf sheath**, **Peduncle**; (2)	OsZIP6 (Os05g0164800) (Kavitha et al., 2015)	(1)Main-expressed in **root**, **shoot**, **seed**, **leaf**; (2) Expression in root induced by Low-Fe, Low-Zn and Low-Mn	Fe/Cd/Co uptake and transport	AtZIP6 (AT2G30080)	**AtZIP6**: △zrt1△zrt2 (N), △fet3△fet4 (N), △ctr1△ctr3 (N), △smf1 (Y) (Zheng et al., 2018)	**Conserved gene function in Poaceae**; Fe/Cd uptake and transport
SiZIP12	(1)**FS-Panicle**, **FS-Root**; (2)Up-regulated expression in root induced by Low-K and High-K, inhibited by Low-Fe and High-Fe	OsIRT1 (Os03g0667500) (Lee and An, 2009; Nakanishi et al., 2006)	(1)Main-expressed in **root epidermis (the inner layer of the cortex, and the stele),** stems (companion cells) and **seed**; (2) Expression in root induced by Low-Fe and Low-Cu	(1)Fe uptake in root under Fe limitation; (2) Cd uptake in root and transport to shoot; (3) affecting the Zn and Fe content in grains	AtIRT2 (AT4G19680) AtIRT1 (AT4G19690) (Fe/Zn/Cd uptake in root)	**OsIRT1**: △fet3△fet4 (Y), △fet1△fet3△fet4(Y), △zrt1△zrt2 (N), △smf1 (N), △ctr1 (N) **AtIRT2**: △zrt1△zrt2 (Y), △fet3△fet4 (Y), △smf1 (N) **AtIRT1**: △zrt1△zrt2 (Y), △fet3△fet4 (Y), △ctr1△ctr3 (N), △smf1 (Y) (Zheng et al., 2018)	**Incomplete conservation of gene function**; Besides Fe/Cd uptake in root and transport to shoot, may participate in the grain Fe accumulation
SiZIP13	(1)**FS-Root**; (2)Up-regulated expression in root induced by Low-K, High-K and High-Fe	OsIRT2 (Os03g0667300) (Nakanishi et al., 2006)	(1)Main-expressed in **root epidermis (the inner layer of the cortex, and the stele)**; (2) Expression in root induced by Low-Fe	(1)Fe uptake in root under Fe limitation; (2) Cd uptake in root and transport to shoot;	AtIRT2 (AT4G19680) AtIRT1 (AT4G19690) (Fe/Zn/Cd uptake in root)	**OsIRT2**: △fet3△fet4 (Y), △smf1 (N), △ctr1 (N) **AtIRT2**: △zrt1△zrt2 (Y), △fet3△fet4 (Y), △smf1 (N) **AtIRT1**: △zrt1△zrt2 (Y), △fet3△fet4 (Y), △ctr1△ctr3 (N), △smf1 (Y) (Zheng et al., 2018)	**Conserved gene function in Poaceae**; Fe/Cd uptake and transport
SiZIP14	(1)**FS-Leaf, Leaf sheath**; (2)Up-regulated expression in leaf induced by Low-Cd, Moderate-Cd, Low-K and High-K	OsZIP2 (Os03g0411800)	(1)Main-expressed in root, **leaf**, Inflorescence and seed;	NA	AtZIP11 (AT1G55910)	**AtZIP11**: △zrt1△zrt2 (Y), △fet3△fet4 (N), △ctr1△ctr3 (N), △smf1 (N) (Zheng et al., 2018)	may be involved in the transport and distribution of Zn in different tissues

^a^ FS, filling stage; HS, heading stage; PS, Pollination stage; SS, Seedling stage; BS, boot stage. Those tissues where the orthologs were specifically expressed in foxtail millet and rice are bolded.

**Figure 9:**
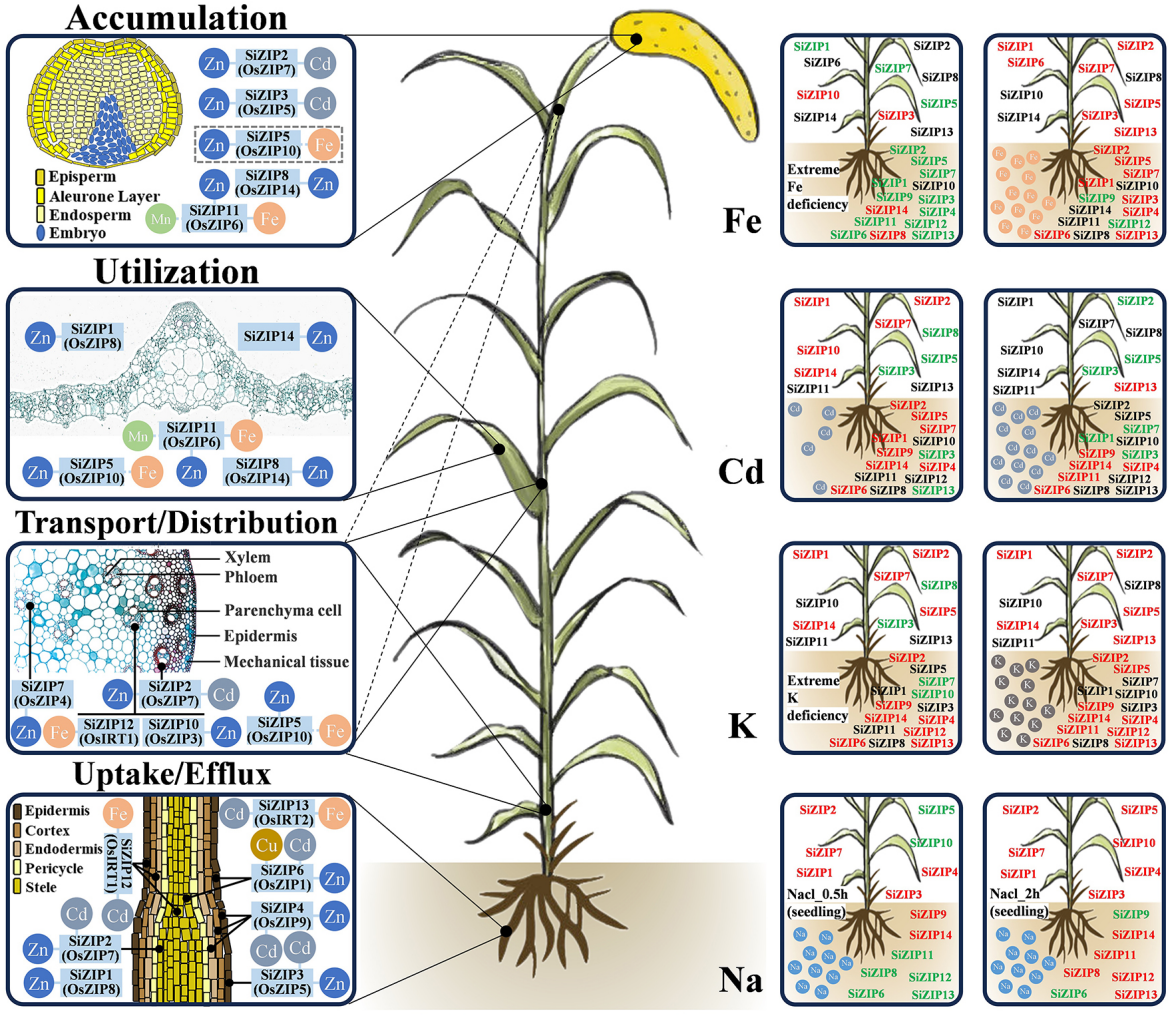
The schematic diagram of predicted functions and response to metal ions of SiZIPs in foxtail millet. Functional predictions of SiZIPs are derived from homology comparisons, expression profiling and previous studies. SiZIPs and their conserved orthologs in rice are labeled together. The left side shows the main functions involved by SiZIPs, while the right side shows the response of SiZIPs to different metal ions. Red represents up-regulation and green represents downregulation. Except for Na treatment, the expression levels of SiZIPs between aboveground and underground parts were distinguished.

Based on the homology relationships and expression patterns of *ZIPs* between foxtail millet, rice, and *Arabidopsis*, we predicted the functions of most *SiZIP* genes. Ultimately, we created a preliminary model diagram of the *SiZIP* genes in foxtail millet, integrating gene function predictions and expression characteristics in response to metal ions (**Figure 9**). According to the expression levels of the genes in different tissues and previous studies, the functions of the *SiZIP* genes were mainly classified into four categories: 1) absorption and efflux of metal ions such as Zn, Fe, and Cd in roots; 2) loading, unloading, and distribution of metal ions in stems; 3) intracellular homeostasis and utilization of metal ions in tissues and organs like leaves; 4) accumulation of metal ions in grains. The localization of conserved *SiZIP* genes in different tissues and cells was also depicted as precisely as possible, such as *SiZIP2* (*OsZIP7*) likely localized in the root stele, and *SiZIP7* (*OsZIP4*) potentially positioned in the stem or phloem of leaf axillary meristems. Additionally, the expression changes of *ZIP* genes under non-stress conditions of Fe, Cd, K, and Na were also characterized. In addition to having significant responses to different concentrations of Fe and Cd, the *SiZIP* genes also exhibited general responses to K and Na, which was likely closely related to the complex synergistic and antagonistic relationships between different metal ions and their transporters.

## 4 Discussion

Trace mineral elements such as Fe and Zn are crucial for crop growth, development, quality formation, and human health (Pushnik et al., 1984; Broadley et al., 2012; Pinson et al., 2015; Wang et al., 2024), while excessive accumulation of heavy metals like Cd in crops can easily enter the human body through the food chain, leading to severe health issues (Pan et al., 2016; Yang et al., 2021c). Numerous studies have confirmed that essential trace elements like Zn and Fe, and harmful heavy metals like Cd, are simultaneously absorbed, transported, and accumulated by crops due to their similar geochemical behaviors (Zheng et al., 2018). The ZIP family, as an important metal ion transporter in crops, is directly responsible for the absorption, transport, and distribution of metal ions such as Zn, Fe, and Cd (Eide, 2006). Therefore, they can serve as potential targets to improve the absorption and utilization efficiency of Zn and Fe in crops, ensure food security, and improve nutritional quality. Although *ZIP* genes have been reported in Arabidopsis, rice, wheat and other crops, little is known about them in foxtail millet (Zheng et al., 2018; Li et al., 2021). In this study, we identified 14 *ZIP* genes in foxtail millet, which did not show significant differences in number compared to monocot grasses like sorghum (14), maize (12), rice (13), and dicotyledonous plants like Arabidopsis (15) and potato (12). Phylogenetic analysis revealed that *ZIP* genes from these species were mixedly distributed into four class, indicating that the formation of the ZIP family predates the divergence of monocots and dicots (**Figure 1**). Although the number of *ZIP* genes in different species was similar, except for the relatively conserved *ZIP* genes in Class I across monocots and dicots, *ZIP* genes in other class showed significant uneven expansion. Unique gene clusters formed in Class III and Class IV, such as OC8-1 to OC8-4, were specific to monocots, which may be an important reason for the significant difference in Zn and Fe absorption between monocots and dicots (**Table 2**) (Zheng et al., 2018). Through an in-depth assessment of phylogenetic relationships, we clarified the orthologous relationships of *ZIP* genes. *ZIP* genes in orthologous gene clusters across multiple species may retain the most basic functions from their ancestor before the divergence of monocots and dicots. On the other hand, paralogous genes formed after divergence have greatly enriched the functional diversity of the *ZIP* gene family, playing an important role in the environmental adaptability of different species.

The expansion and functional divergence of gene families often rely on early duplication events and the emergence of novel genes and functions that follow thereafter (Yang et al., 2021b). More than 50% (8/14) of the genes in the foxtail millet ZIP family were involved in gene duplication events, forming two pairs of TDs and two pairs of SDs (**Supplementary Figure S1**), which confirmed the contribution of duplication events to the expansion of the foxtail millet *ZIP* gene family. Although the Ka/Ks values of all duplicated gene pairs were less than 1, the TD gene pairs had relatively higher values, indicating that under the premise of conserving important functions, new genes generated by TD may be more crucial for the functional divergence of the *SiZIP* gene family, similar to reports in other species (Gao et al., 2022). In addition to differences in sequences, duplicated gene pairs also exhibited new variations in gene structure, protein conserved domains, and transmembrane regions. *SiZIP5* and *SiZIP1* (SD1), *SiZIP7* and *SiZIP10* (SD2) had deletions in conserved motifs (**Figure 2**), while *SiZIP7* and *SiZIP10* (SD2), *SiZIP3* and *SiZIP4* (TD1), and *SiZIP12* and *SiZIP13* (TD2) showed variations in the number of TM domains (**Table 1**). As important transmembrane transporters, the TM regions are crucial for protein function (Yang et al., 2022). Moreover, the duplicated gene pairs also showed significant differences in tissue expression patterns (**Figure 5**, **Supplementary Figure S2**). The *SiZIP2* gene in SD1 was highly expressed in panicles and roots during multiple stages of foxtail millet growth, while *SiZIP5* was mainly expressed in leaves. In TD1, *SiZIP3* was specifically expressed in panicles, while *SiZIP4* was highly expressed in germinating seeds. In TD2, *SiZIP13* was specifically expressed in roots, while *SiZIP12* was highly expressed in both roots and panicles. Additionally, we found that the responses of these genes to different metal ions have also changed. For example, the expression level of *SiZIP7* in SD2 was significantly induced by high Fe, while *SiZIP10* did not respond. In conclusion, while gene duplication drives the expansion of the SiZIP family, the changes in sequence, protein structure, and expression characteristics of the newly generated genes directly promote the functional divergence of the SiZIP family.

The spatiotemporal expression characteristics and responses to different stresses of members within the same gene family often exhibit significant differences, providing fundamental information for understanding the functions of important genes (Yang et al., 2021b). The expression patterns of *SiZIP* genes showed clear temporal and spatial specificity throughout the growth period of foxtail millet (**Figure 5**, **Supplementary Figure S2**). Specifically, *SiZIP9*, *SiZIP4*, and *SiZIP6* were highly expressed in germinating seeds and seedlings, likely participating in seed germination and seedling growth by regulating the absorption and transport of Zn, Fe, and other ions. In contrast, *SiZIP8* and *SiZIP5* were primarily expressed in the panicles, stems, and leaves during the later stages of foxtail millet growth and development, potentially involved in the transport and redistribution of metal ions. Furthermore, some *SiZIP* genes exhibited tissue-specific expression patterns, such as *SiZIP13*, *SiZIP14*, *SiZIP10*, and *SiZIP3*, which were specifically expressed in roots, leaves, stems, and panicles, respectively, indicating more specialized functions. *ZIP* genes are not only responsible for the migration of Zn and Fe in plants but also for the absorption and transport of other metals (Zheng et al., 2018).

Most *SiZIP* genes responded to different metal ion stresses, including Fe, Cd, K, and Na (**Figures 6**, **7**). Over 50% of *SiZIP* genes (8/14) were significantly upregulated under high Fe stress, similar to the response pattern of *OsZIP* in rice (Zheng et al., 2018). *SiZIP6* and *SiZIP4* were significantly upregulated under Cd stress, which was consistent with the response pattern of their orthologs *OsZIP1* and *OsZIP9* (Liu et al., 2019; Huang et al., 2020; Tan et al., 2020). Interestingly, *SiZIP* genes show widespread responses to K and Na, and some of them exhibited inconsistent response patterns with Fe, such as *SiZIP12* and *SiZIP9*. After Cd treatment, significant changes occurred in the accumulation of Fe, Ca, K, and Na in the roots and aboveground tissues of foxtail millet seedlings. At the same time, there was a significant correlation between *SiZIP* gene expression and metal ion content, especially with Fe and K (**Supplementary Figure S3**). These results suggested that *SiZIP* genes, in addition to their previously reported role in transporting divalent metal ions, also had indirect synergistic or antagonistic relationships with the transport of monovalent metal ions such as Na and K. Additionally, some *SiZIP* genes, such as *SiZIP1* and *SiZIP3*, were upregulated under cold and drought stress. These genes may repair oxidative damage and maintain membrane stability through the transport of metal ions, thus resisting plant damage caused by stress (Coleman, 1998; Cakmak, 2000). Additionally, a large number of cis-acting elements involved in stress response and tissue expression specificity were found in the promoter regions of multiple *SiZIPs* (**Figure 4**). These elements may be related to the expression levels of *SiZIPs*. For example, seed-specific expression elements were found in the upstream of *SiZIP4* and *SiZIP6*, which is consistent with the specific high expression of these two genes in germinating seeds (**Supplementary Figure S2**). The upstream of *SiZIP12* contains four low-temperature response elements, which may be related to its rapid up-regulated expression under low-temperature stress (**Figure 7**). The multiple stress response elements upstream of *SiZIP13* may be closely related to its extensive response to various abiotic stresses. A large number of studies have confirmed that transcription factors such as MYB affect biotic and abiotic stress responses by regulating the expression of metal transport genes (Liu et al., 2024). Although we did not find cis-acting elements directly related to metal ion transport in the upstream of *SiZIPs*, the presence of multiple MYB transcription factor binding elements may be related to their metal transport. In general, we have found the possible associations between multiple cis-acting elements in the upstream of *SiZIPs* and their expression levels. A more in-depth and accurate regulatory mechanism needs to be further verified through experiments.

Analyzing genetic variations in important functional genes in germplasm resources is an important means to clarify their value for breeding improvement (Liu et al., 2022). We identified a total of 1718 SNPs and Indels distributed across *SiZIP* genes (including upstream and downstream regions) in 360 foxtail millet genotypes, including 235 mutations located in promoter regions and 54 non-synonymous SNPs (**Table 3**). Further association analysis between grain metal ion content and variation sites revealed 102 SNPs significantly associated with Fe, Zn, Cd, and other metal ion contents (unpublished data). Additionally, the expression levels of *SiZIP* genes showed significant differences in grains of different foxtail millet genotypes (**Supplementary Figure S4**). The above results confirmed the significant genetic selection potential of *SiZIP* genes in the improvement of foxtail millet quality.

During the domestication process of gramineae crops, there has been a large-scale convergent selection, with multiple important genes including *OsKRN2*, *TaGS-D1*, *TaCKX2*, *TaTGW6*, and *TaCWI* reported to regulate traits such as yield in maize, rice, and wheat through similar pathways (Zhang et al., 2011, 2014; Jiang et al., 2015; Hanif et al., 2016; Chen et al., 2022; He et al., 2024). Meanwhile, by comparing the orthologous relationships and expression characteristics of functional genes in rice, several important genes related to wheat and foxtail millet yield have been discovered (Yang et al., 2021a; Liu et al., 2022). These findings confirmed that through homologous comparison, expression analysis, and in-depth research on functional genes of leading model species, functional genes in less-studied secondary crops such as foxtail millet can be rapidly identified. Based on this, we combined the research on *ZIP* genes in rice and Arabidopsis, and predicted the function of *ZIP* genes in foxtail millet under the premise of fully considering the expression characteristics of orthologous genes (**Table 4**). Multiple *SiZIP*s and their orthologous genes exhibited highly similar expression patterns in foxtail millet and rice. *SiZIP7* was specifically highly expressed in stems and peduncles of foxtail millet, and its orthologous gene *OsZIP4* has been proven in rice to be highly expressed in the vascular bundle system of stem nodes and axillary meristems to regulate the preferential distribution of Zn in different organs (Mu et al., 2021), suggesting that *SiZIP7* may have similar functions in foxtail millet. *SiZIP13* was specifically expressed in foxtail millet roots, while its orthologous gene *OsIRT2* was localized in the stele and epidermis of rice roots and was responsible for Fe/Cd uptake in roots (Nakanishi et al., 2006). *SiZIP3* and its orthologous gene *OsZIP5* were both specifically highly expressed in panicles, and *OsZIP5* has been shown to be involved in the regulation of Zn homeostasis, indicating that the functions of *SiZIP3*/*OsZIP5* may be relatively conserved (Tan et al., 2020). *OsZIP1*, as a well-documented important metal efflux transporter that was specifically expressed in the roots of seedlings, exhibited a similar expression pattern in its foxtail millet ortholog *SiZIP6*, suggesting that they may share similar functions. *SiZIP1* was highly expressed in the leaves and roots of foxtail millet, and overexpression of its orthologous gene *OsZIP8* with the same expression characteristics resulted in a decrease in Zn content in the shoots and an increase in Zn content in the roots of transgenic rice plants, suggesting that *SiZIP1* may be involved in the distribution of Zn in the shoots similar to *OsZIP8* (Lee et al., 2010b). We found that at least 8 *SiZIP* genes have similar expression patterns with their rice orthologous genes, and the functions of these genes may be highly conserved between foxtail millet and rice, similar to the previous reports of hundreds of genes being convergently selected in maize and rice (Chen et al., 2022). In addition to these conserved genes, a few genes showed inconsistent expression patterns, which may have some foxtail millet-specific functions. Overall, based on the expression patterns, orthologous relationships, and previous reports of these genes, we have predicted the functions of *SiZIP* genes as much as possible and classified them into four categories: root absorption/efflux, stem transport/distribution, utilization in tissues such as leaves, and grain development and accumulation (**Figure 9**). Some *SiZIP* genes were only involved in one of these processes, while others played roles in multiple processes. In general, although the functions of individual *SiZIP* genes were not studied in depth in this study, and there is a lack of relevant results on their response to Zn, a basic research framework for *SiZIP* genes in foxtail millet has been constructed through the combined strategy of systematic characterization, homologous comparison, and expression characteristic analysis. More in-depth functional studies will be the focus of our next work.

## 5 Conclusion

In this study, 14 ZIP transporters were accurately identified and characterized in foxtail millet. Based on the phylogenetic relationships of ZIPs from six species, SiZIPs were divided into four evolutionary branches and their homologous relationships with ZIPs of staple crops were clarified. We demonstrated that gene duplication and subsequent multi-level variations jointly promote the expansion and functional differentiation of the ZIP family in foxtail millet. These *SiZIP* genes showed abundant spatiotemporal expression characteristics in different tissues of foxtail millet during the whole growth period, and exhibited positive responses to Fe, Cd, K, Na, drought and cold stresses. It’s indicated that they might be extensively involved in the transport of metal ions and osmotic regulation under abiotic stresses. Additionally, we analyzed the sequence variation and expression variation of SiZIP in different genotypes of foxtail millet, and evaluated its genetic potential in improving foxtail millet quality. Finally, we predicted the function of SiZIP gene in foxtail millet and preliminarily drew its functional pattern diagram by integrating homologous comparison, expression analysis and previous studies. This study will lay the foundation for further functional research on *SiZIPs*, and will also contribute to the elucidation of the molecular mechanisms underlying metal ion transport and accumulation in foxtail millet and the quality improvement.

## Data Availability

The original contributions presented in the study are included in the article/**Supplementary Material**. Further inquiries can be directed to the corresponding author/s.
