# Supplementary material for: An integration of genome-wide survey, homologous comparison and gene expression analysis provides a basic framework for the ZRT, IRT-like protein (ZIP) in foxtail millet
Source: Front Plant Sci. 2024 Sep 5;15:1467015. doi: 10.3389/fpls.2024.1467015 (PMC11410603; doi:10.3389/fpls.2024.1467015)
Supplement: Supplementary file 1 [file DataSheet1.docx]

**Supplementary Figure legends**

**Supplementary Figure S1. Distribution of ZIP genes on chromosomes in foxtail millet.** Tandem duplication (TD), segmental duplication (SD) and the Ka/Ks values are displayed by blue shadows, red lines and the numbers in black boxes, respectively.

**Supplementary Figure S2. The spatiotemporal expression patterns of *SiZIPs* in multiple tissues during whole growth period in foxtail millet.** The expression datasets are obtained from foxtail millet multi-omics database (MDSi). The visualization is achieved by TBtools. The orange and green boxes represent TD gene pairs and SD gene pairs, respectively.

**Supplementary Figure S3. The change of ionome in foxtail millet seedlings and correlation with *SiZIP* expression level under Cd treatment.** (A) The internal value indicates the content of metal ions and the external value indicates the expression level of *SiZIP* gene; (B) The heatmap shows the correlation between *SiZIP* expression level and ion content, with red indicating a positive correlation and blue indicating a negative correlation. The Pearson correlation coefficient is also listed. Cd1, 5 µM; Cd2, 10 µM; Cd3, 30 µM.

**Supplementary Figure S4. The expression levels of *SiZIPs* in grains of 360 foxtail millet genotypes at filling stages**. The Y-axis represents the TPM value based on RNA-seq and the X-axis represents the different genotypes.
