# Supplementary figures and images for: An integration of genome-wide survey, homologous comparison and gene expression analysis provides a basic framework for the ZRT, IRT-like protein (ZIP) in foxtail millet
Source: Front Plant Sci. 2024 Sep 5;15:1467015. doi: 10.3389/fpls.2024.1467015 (PMC11410603; doi:10.3389/fpls.2024.1467015)

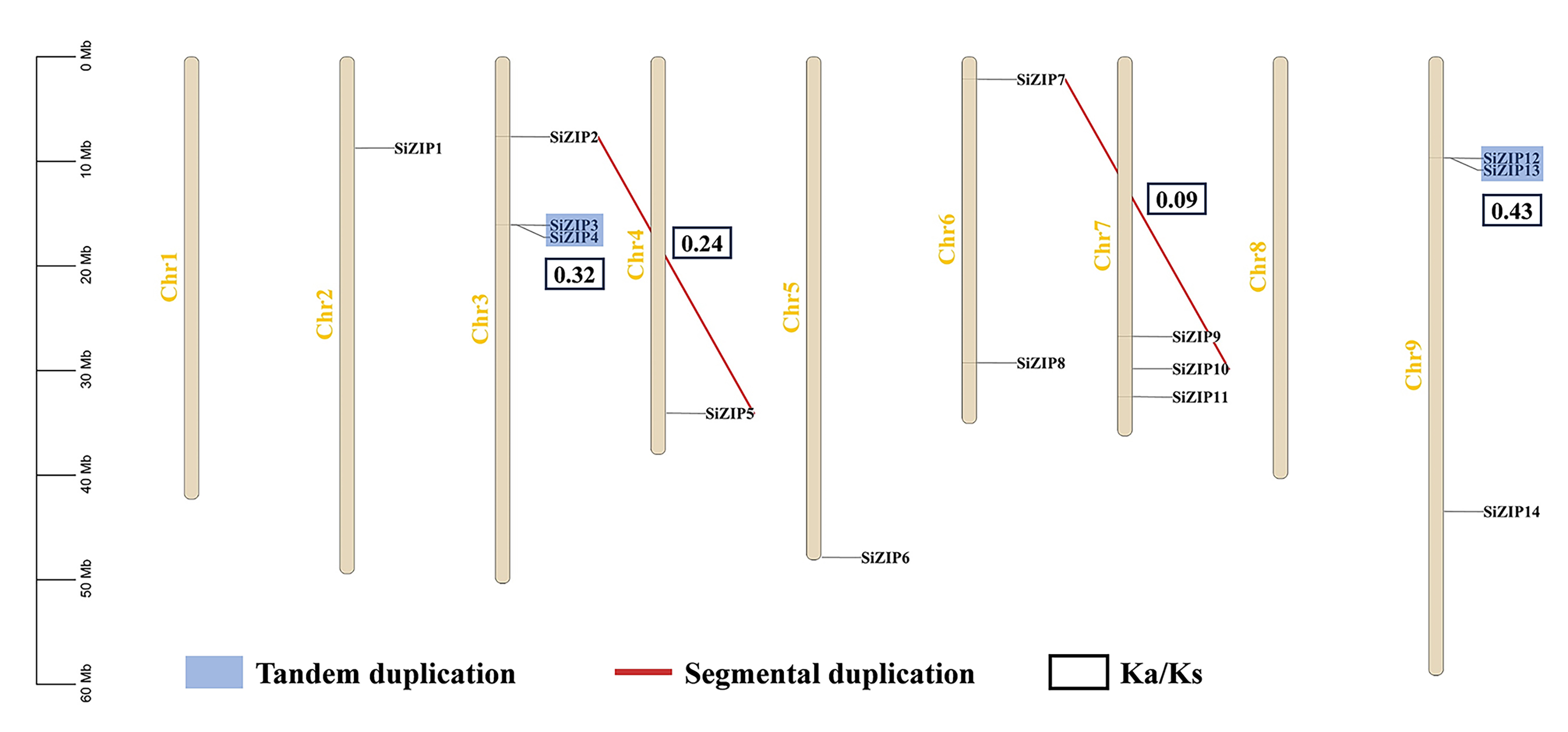

Supplement: Supplementary file 2 [file Image1.jpeg]

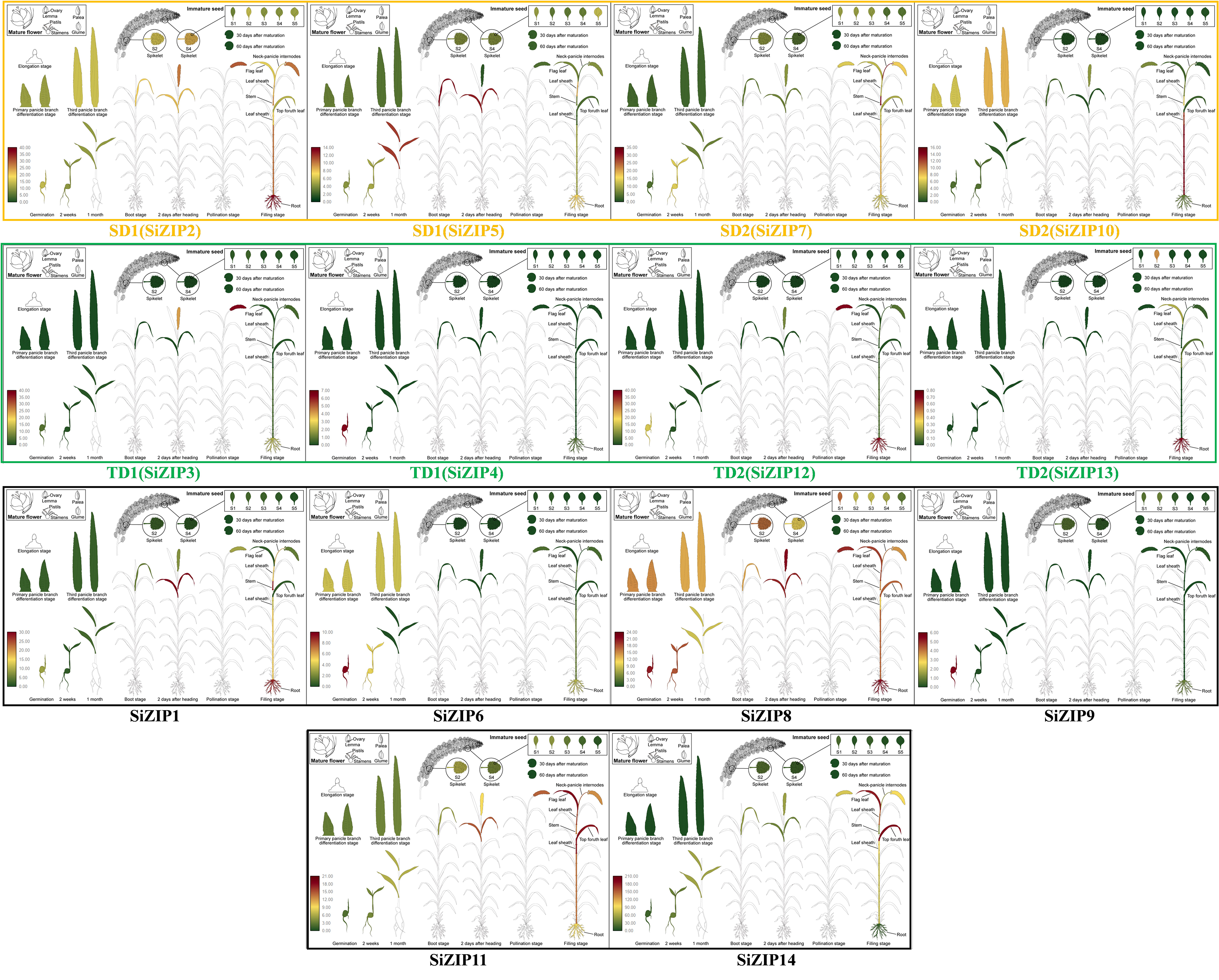

Supplement: Supplementary file 3 [file Image2.jpeg]

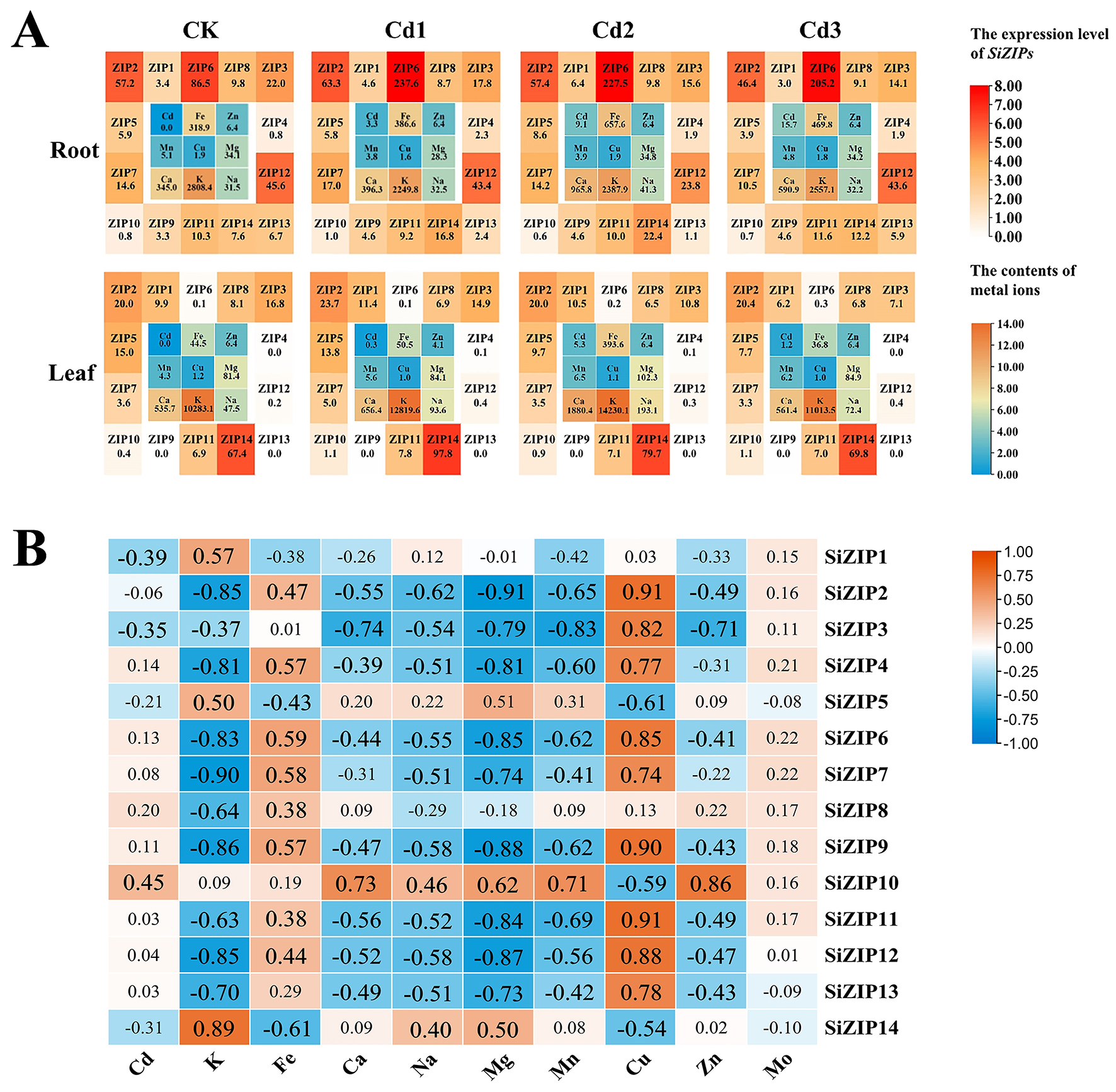

Supplement: Supplementary file 4 [file Image3.jpeg]

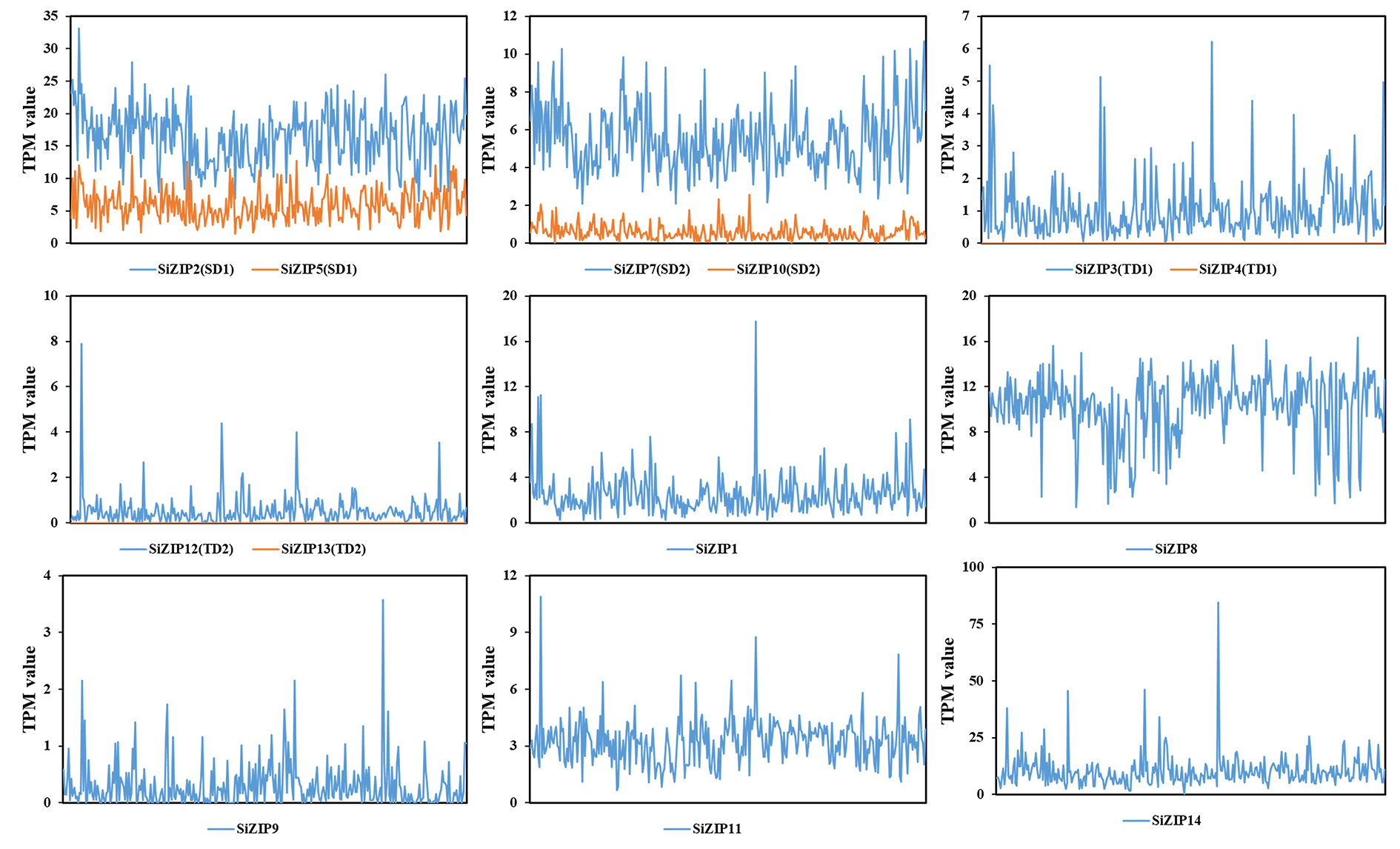

Supplement: Supplementary file 5 [file Image4.jpeg]
